# Damage Models for Soft Tissues: A Survey

**DOI:** 10.1007/s40846-016-0132-1

**Published:** 2016-06-08

**Authors:** Wenguang Li

**Affiliations:** School of Engineering, University of Glasgow, Glasgow, G12 8QQ UK

**Keywords:** Soft tissue, Damage, Continuum damage mechanics, Fiber-reinforced material, Constitutive law

## Abstract

Damage to soft tissues in the human body has been investigated for applications in healthcare, sports, and biomedical engineering. This paper reviews and classifies damage models for soft tissues to summarize achievements, identify new directions, and facilitate finite element analysis. The main ideas of damage modeling methods are illustrated and interpreted. A few key issues related to damage models, such as experimental data curve-fitting, computational effort, connection between damage and fractures/cracks, damage model applications, and fracture/crack extension simulation, are discussed. Several new challenges in the field are identified and outlined. This review can be useful for developing more advanced damage models and extending damage modeling methods to a variety of soft tissues.

## Introduction

Soft tissue is a general term that refers to various groups of cells in the human body. All tissues in the body that are neither bones nor organs are considered soft tissues. Soft tissues can be divided into connective tissues, such as tendons, ligaments, fascia, skin, fibrous tissues, fat, and synovial membranes, and non-connective tissues, such as muscles, nerves, and blood vessels [1]. The major physiological functions of soft tissues are to connect, support, and surround organs and other structures of the body. Some soft tissues, namely arterial smooth muscle, skeletal muscle and cardiac muscle, are stretched to generate a passive tension, but can also contract to generate an active tension. However, other soft tissues such as ligaments, tendons, and skins can be stretched to have passive tension only.

**Fig. 1:**
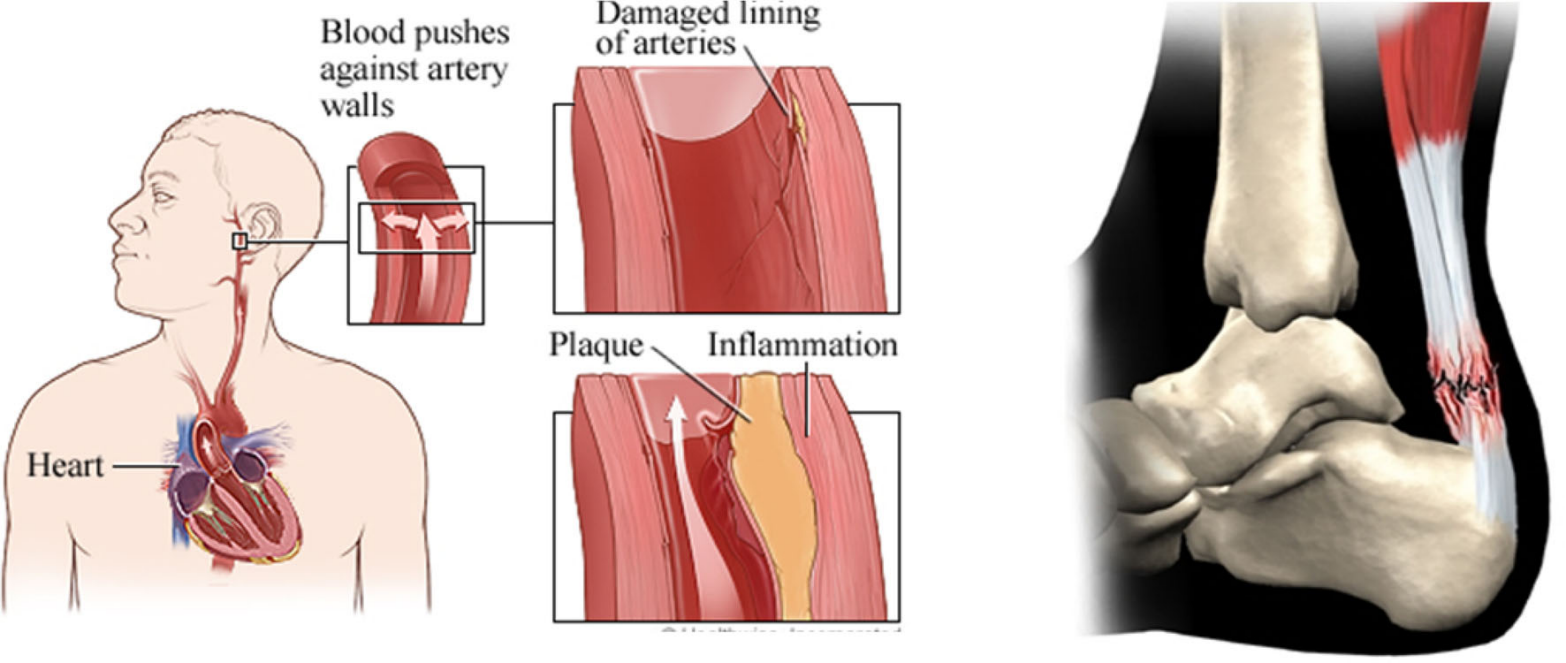
Artery inner wall is damaged by high blood pressure of heart and achilles tendon is ruptured by accident. **a** artery (http://www.webmd.com/hypertension-high-blood-pressure/how-high-blood-pressure-damages-arteries) and **b** tendon (http://www.methodistorthopedics.com/achilles-tendon-problems)

Soft tissues, especially arterial smooth muscles, ligaments, and tendons in joints of the human body can be injured or damaged by disease or excessive force applied during exercise, accidents, or surgery. For example, the human arterial inner wall can be damaged by high blood pressure, with subsequent plaque development. A tendon can be damaged or ruptured, as shown in Fig. 1. Knowledge of soft tissue damage, injury, or failure behavior is very helpful for artificial soft tissue design and fabrication.

In addition to experiments, finite element analysis (FEA) is extensively used for soft tissue damage characterization when the tissue is in vivo state for surgery [2]. However, the damage models are based on existing in vitro measurements. Unfortunately, soft tissues usually exhibit nonlinear, heterogeneous, anisotropic, and viscoelastic behavior, making their damage modeling difficult. Thus, the understanding, physical description, and modeling of damage and failure in soft tissues have presented a tough challenge. It is necessary to assess existing soft tissue damage models and to take a forward look for further study.

Damage models developed for soft tissues can be traced back to the 1970s. These models can be divided into three categories: (1) deterministic models, in which a pseudo-elastic strain energy function with a few parameters or a strain energy function with a few damage variables of continuum damage mechanics is used to account for the softening/damage effect, and soft tissue can be either isotropic or anisotropic; (2) probabilistic models, in which the fiber recruitment effect, probabilistic damage process, or both are involved; in some models a damage variable of continuum damage mechanics is used; (3) microstructure-based damage models of collagen fibers, in which the individual collagen fibril damage behavior is characterized and then integrated into the whole tissue level. The last two kinds of model are anisotropic only. These damage models are summarised in Table 1.

**Table 1:** Summary of damage models for soft tissues

References	Type	Tissue	Tissue structure	Damage	Features
Miehe [7]	Deterministic	Rubber-like material	Isotropic rubber matrix and filled particles	Bonds between rubber matrix and filled particles	(1) Isotropic, compressible, (2) strain energy function, (3) damage variables for discontinuous and continuous damage mechanisms
Ogden and Roxburgh [3]	Deterministic	Rubber-like material	Isotropic rubber matrix and filled particles	Bonds between rubber matrix and filled particles	(1) Isotropic, incompressible, (2) strain energy function, (3) two parameters related to softening effect
Volokh [16]	Deterministic	Rubber-like material, AAA	Isotropic matrix	Matrix	(1) Isotropic, incompressible, (2) one parameter related to softening effect
Alastrué et al. [39]	Deterministic	Soft tissues with fibers	Isotropic matrix material and collagen fibers	Matrix and fibers	(1) Anisotropic, compressible, (2) strain energy function for matrix and fibers, (3) four damage variables
Volokh [24]	Deterministic	Artery	Isotropic matrix material and collagen fibers	Matrix and fibers	(1) Anisotropic, incompressible, (2) Holzapfel’s strain energy function, (3) three parameters related to softening effect
Peña and Doblaré [33] and Garcia et al. [35]	Deterministic	Soft tissues with fibers	Isotropic matrix material and collagen fibers	Matrix and fibers	(1) Anisotropic, compressible, (2) strain energy function for matrix and fibers, (3) four parameters related to softening effect, (4) extension of work by Ogden and Roxburgh [3]
Calvo et al. [37] and Martins et al. [38]	Deterministic	Vaginal and rectus sheath tissue	Isotropic matrix material and collagen fibers	Matrix and fibers	(1) Anisotropic, compressible, (2) strain energy function for matrix and fibers, (3) two damage variables
Li and Robertson [31, 32]	Deterministic	Cerebral arterial tissue	Collagen fibers and elastin	Elastin	(1) Anisotropic, incompressible, (2) strain energy function for elastin and fibers, (3) three damage variables
Ehret and Itskov [26] and Itskov and Ehret [83]	Deterministic	Soft tissue with fibers	Isotropic matrix material and collagen fibers	Fibers	(1) Anisotropic, incompressible, (2) poly-convex strain energy function for matrix and fibers, (3) softening effect considered by decreasing fiber initial stiffness
Volokh [25]	Deterministic	Artery, AAA	Isotropic matrix material and collagen fibers	Matrix and fibers	(1) Anisotropic, incompressible, (2) Hozapfel’s strain energy function, (3) three strain energy limiters and sharpness factors
Peña et al. [27] and Balzani et al. [28]	Deterministic	Soft tissue with fibers	Isotropic matrix material and collagen fibers	Matrix and fibers	(1) Anisotropic, compressible, (2) strain energy function for matrix and fibers, (3) discontinuous and continuous damage mechanisms considered
Maher et al. [30]	Deterministic	Soft tissue with fibers	Isotropic matrix material and collagen fibers	Matrix and fibers	(1) Anisotropic, compressible, (2) strain energy function for matrix and fibers, (3) two damage variables, (4) plastic effect is included
Marini et al. [11]	Deterministic	AAA	Collagen fibers and elastin	Fibers	(1) Isotropic, compressible, (2) strain energy function, (3) one damage variable
Waffenschmidt et al. [42]	Deterministic	Artery	Collagen fibers and matrix material	Fibers	(1) Anisotropic, compressible, (2) local free energy function, (3) non-local damage variable and ordinary damage variable
Chu and Blatz [43]	Probabilistic	Cat mesentery	Collagen fibers, elastin, reticulum	Fibers	(1) Isotropic, incompressible, (2) Ogden’s strain energy function (3) probability function for stress, (4) no damage variable
Liao and Belkoff [52]	Probabilistic	Ligaments	Collagen fibers and elastin	Fibers	(1) Anisotropic, incompressible, (2) linear elastic stiffness, (3) fiber recruitment effect
Natali et al. [59]	Probabilistic	Tendons	Isotropic matrix material and collagen fibers	Fibers	(1) Anisotropic, compressible, (2) strain energy function for matrix and fibers, (3) fiber recruitment effect, (4) one damage variable
De Vita and Slaughter [53]	Probabilistic	Medial collateral ligaments	Collagen fibers and elastin	Fibers	(1) Anisotropic, incompressible, (2) linear elastic stiffness, (3) fiber recruitment effect
Guo and De Vita [54]	Probabilistic	Medial collateral ligaments	Collagen fibers and elastin	Fibers	(1) Anisotropic, incompressible, (2) linear elastic stiffness, (3) fiber recruitment effect, (4) one damage variable
Schmidt et al. [41]	Probabilistic	Arterial walls	Collagen fibers and isotropic matrix material	Fibers	(1) Anisotropic, compressible, (2) strain energy function for matrix and fibers, (3) one damage variable related to probabilistic proteoglycan bridge damage of collagen fibrils
Gasser [60]	Microstructure	AAA	Collagen fibers and elastin	Fibers	(1) Anisotropic, incompressible, (2) microstructure strain energy function for fiber, (3) collagen recruitment effect, (4) damage variable and viscoelastic effect

The rest of this paper is organized as follows. Section 2 reviews damage models for isotropic soft tissues as they provide a basis for damage modeling, especially the model for rubber-like materials proposed by Ogden and Roxburgh [3]. In Sect. 3, a survey of damage models for anisotropic soft tissues, including the arterial wall, ligaments, and tendons, is given. Discussions and a few challenges for damage models are highlighted in Sect. 4. Finally, concluding remarks are given in Sect. 5.

## Damage Models for Isotropic Materials

### Rubber-Like Materials

The concept of continuum damage mechanics has been increasingly applied to predict damage in soft tissues. Damage mechanics involves the engineering predictions of the initiation, propagation, and fracture in a material using state variables, which represent the effects of damage caused from thermal or mechanical loading or aging on the stiffness and remaining life of the material [4, 5]. The state variables may be measurable variables or other physical variables.

**Fig. 2:**
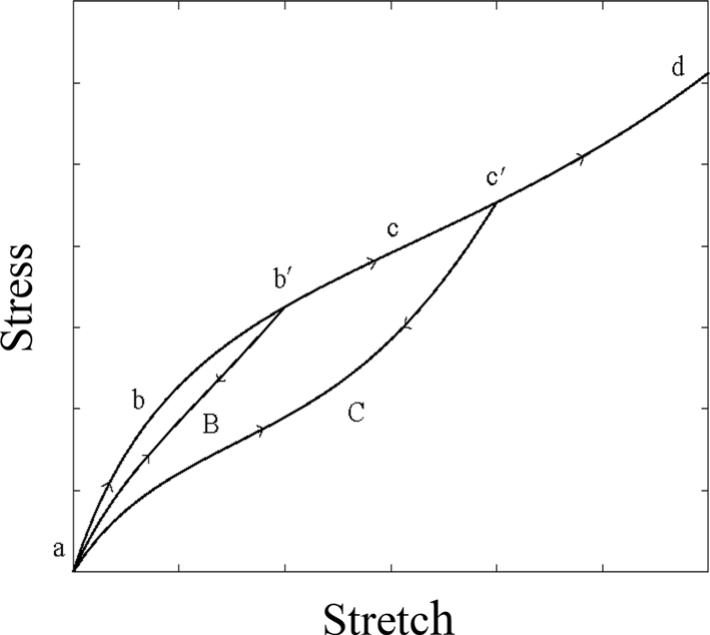
Sketch of loading–unloading paths exhibiting the Mullins effect in simple tensile test under cyclic loading, adapted from [3]. Note that because unloading curve comes back to the origin, there is no plastic deformation in material

The damage of soft isotropic materials is modeled in continuum damage mechanics as follows. A specimen of rubber or soft tissue often exhibits the Mullins effect in a simple tensile test under a cyclic load, as sketched in Fig. 2. Initially, the sample is loaded to *b*′ from *a*; the loading path is *abb*′. If the sample is unloaded from *b*′, then the unloading path is *b*′ *Ba*. If the sample is reloaded to point *c*′, then the loading path will be *aBb*′ *cc*′ and the new unloading path will be *c*′ *Ca*. Such a path pattern is repeated until a total failure occurs in the sample under cyclic loading. This phenomenon is named the Mullins effect or stress softening.

The Mullins effect is interpreted as damage occurring in the sample at the microscopic level. This may be due to the bonds between the filler particles and the molecular chains being broken for rubber or collagen fiber being broken for soft tissues.

Since the lengths of chain links in rubber and collagen fibers vary, and they can break at different stretches as the damage process proceeds. Additionally, after damage, the reloading path is identical to the previous unloading path. This suggests that the energy consumed by damage is irrecoverable.

For a general biaxial simple tensile test, the following specific pseudo-elastic strain energy function was proposed by Ogden and Roxburgh [3] for an incompressible rubber-like material:

1 $$ \psi \left( {\lambda_{1},\lambda_{2},\eta } \right) = \eta \tilde{\psi }\left( {\lambda_{1},\lambda_{2} } \right) + \phi \left( \eta \right) $$

where *λ*_1_ and *λ*_2_ are two principal stretches, $\tilde{\psi }\left( {\lambda_{1},\lambda_{2} } \right)$ is an Ogden-type strain energy without damage, expressed as:

2 $$ \tilde{\psi }\left( {\lambda_{1},\lambda_{2} } \right) = \mu \sum\limits_{i = 1}^{3} {\frac{{\mu_{i} }}{{\alpha_{i} }}\left( {\lambda_{1}^{{\alpha_{i} }} + \lambda_{2}^{{\alpha_{i} }} + \lambda_{1}^{{ - \alpha_{i} }} \lambda_{2}^{{ - \alpha_{i} }} - 3} \right)} $$

where *α*_1_, *α*_2_, *α*_3_, *μ*_1_, *μ*_2_, and *μ*_3_ are the material constants without damage and *μ* is the shearing modulus. $\phi \left( \eta \right)$ is the damage function that satisfies the following equation:

3 $$ - \frac{d\phi }{d\eta } = m{\mathbf{erf}}^{ - 1} \left[ {r\left( {\eta - 1} \right)} \right] + \tilde{\psi }\left( {\lambda_{1m},\lambda_{2m} } \right) $$

where *λ*_1*m*_ and *λ*_2*m*_ are the principal stretches for the point where unloading has most recently been initiated from the primary loading path; **erf**^−1^ () is the inverse of the error function; *m* and *r* are positive material constants, where *m* is a parameter used to control the dependence of the damage on the extent of deformation, and *r* is a variable used to indicate the extent of damage relative to the virgin state. The variable *η* is expressed as:

4 $$ \eta = 1 - \frac{1}{r}{\mathbf{erf}}\left[ {\frac{1}{m}\left( {\tilde{\psi }\left( {\lambda_{1m},\lambda_{2m} } \right) - \tilde{\psi }\left( {\lambda_{1},\lambda_{2} } \right)} \right)} \right] $$

where *η* ∈ [0,1] and the error function is ${\mathbf{erf}}\left( x \right) = \tfrac{2}{\sqrt \pi }\int\limits_{0}^{x} {e^{{ - t^{2} }} dt} .$ The boundary condition $\phi \left( 1 \right) = 0$ holds for Eq. (3). After damage, the principal stresses are estimated as:

5 $$ \sigma_{i} - \sigma_{3} = \eta \lambda_{i} \frac{{\partial \tilde{\psi }}}{{\partial \lambda_{i} }}, \quad i = 1,2 $$

For the simple tensile case, *λ* = *λ*_1_, *λ*_2_ = *λ*_3_ = *λ*^−1/2^, and *σ*_2_ = *σ*_3_ = 0. The parameters *μ*, *r*, and *m* can be determined from a series of experimental stress–stretch curves. This model has been extended to the case with residual strain [6]. For industrial rubber, the Ogden model seems better than another damage model [7].

In [8], a general continuum damage mechanics model, the Ogden pseudo-elastic model just mentioned above, and Guo’s elastic model were compared against a series of rubber-like material experimental stress–strain curves under cyclic loads to evaluate model performance. It was shown that Ogden’s pseudo-elastic and Guo’s elastic models are better than the general continuum damage mechanics model.

### Continuum Damage Mechanics Method for Isotropic Materials

In this section, the basic idea for modeling the damage effect in continuum damage mechanics is introduced. Considering an isotropic, homogenous, incompressible rubber-like material, the strain energy function in the damage state, $\psi \left( {{\mathbf{F}},d} \right),$ can be expressed in terms of a strain energy function without damage, $\tilde{\psi }\left( {\mathbf{F}} \right),$ and a scalar damage variable, *d*, as follows [8]:

6 $$ \psi \left( {{\mathbf{F}},d} \right) = \left( {1 - d} \right)\tilde{\psi }\left( {\mathbf{F}} \right) $$

where **F** is the deformation gradient tensor associated with the stress-free state, *d* is a continuous variable used to characterize the damage effect in the material, where *d* ∈ [0,1], *d* = 0, no damage; *d* = 1, complete damage; 0 < *d* < 1, a damage state in between. The second Piola–Kirchhoff stress with damage is calculated as:

7 $$ {\text{S}} = \left( {1 - d} \right)\frac{{\partial \tilde{\psi }\left( {\mathbf{F}} \right)}}{{\partial {\text{E}}}} = \left( {1 - d} \right){\tilde{\text{S}}} $$

where **E** is the Green–Lagrange strain tensor and ${\tilde{\text{S}}}$ is the second Piola–Kirchhoff stress without damage. Usually, the scalar damage variable is an exponential function of the effective strain energy function *α*(*t*) at which damage occurs, i.e.:

8 $$ d\left( {\alpha \left( t \right)} \right) = d_{\infty } \left[ {1 - \exp \left( { - \frac{\alpha \left( t \right)}{\beta }} \right)} \right] $$

where *d*_∞_ and *β* are the model parameters determined in experiments. *α*(*t*) can be determined as follows. Because damage is an irreversible process, the second law of thermodynamics should be applicable. In continuum mechanics, the second law of thermodynamics was expressed as the Clausius–Duhem inequality by Guo and Sluys [8]:

9 $$ - \frac{{d\psi \left( {{\mathbf{F}},d} \right)}}{dt} + {\text{S:}}\frac{{d{\text{E}}}}{dt} \ge 0 $$

Note that:

10 $$ \frac{{d\psi \left( {{\mathbf{F}},d} \right)}}{dt} = \frac{{\partial \psi \left( {{\mathbf{F}},d} \right)}}{{\partial {\text{E}}}}\frac{{d{\text{E}}}}{dt} + \frac{{\partial \psi \left( {{\mathbf{F}},d} \right)}}{\partial d}\dot{d} $$

According to Eq. (6), ${{\partial \psi \left( {{\mathbf{F}},d} \right)} \mathord{\left/ {\vphantom {{\partial \psi \left( {{\mathbf{F}},d} \right)} {\partial d}}} \right. \kern-0pt} {\partial d}}$ can be written as:

11 $$ - \frac{{\partial \left( {{\mathbf{F}},d} \right)}}{\partial d} = \tilde{\psi }\left( {\mathbf{F}} \right) $$

Eventually, Eq. (9) is reduced to the very simple form:

12 $$ \tilde{\psi }\left( {\mathbf{F}} \right)\dot{d} \ge 0 $$

This suggests that the damage process is driven by the strain energy function (thermodynamic force) without damage, $\tilde{\psi }({\mathbf{F}}).$ Accordingly, we can establish a damage criterion based on $\tilde{\psi }\left( {\mathbf{F}} \right)$ from experiments under a series of loads versus time, i.e. $\alpha \left( t \right) = {\text{Max }}\tilde{\psi }\left( {{\mathbf{F}}\left( t \right)} \right),$ which is the maximum strain energy function without damage. When $\tilde{\psi }\left( {\mathbf{F}} \right)$ caused by a certain load profile equals *α*(*t*), damage will occur. This criterion is expressed mathematically as:

13 $$ \phi = \tilde{\psi }\left( {{\mathbf{F}}\left( t \right)} \right) - \alpha \left( t \right) \le 0 $$

If $\phi < 0,$ there is no damage at all; otherwise, if $\phi = 0,$ damage occurs. The variable *α*(*t*) can be used for damage evaluation with time. In this model, the parameters $\tilde{\psi }\left( {\mathbf{F}} \right),$*α*(*t*), *d*_∞_, and *β* need to be determined from a series of experimental stress–stretch curves in various time-dependent loading courses.

Discontinuous and continuous damage evolution models were proposed by Miehe [7] for rubber-like materials based on the Ogden-type strain energy function. The models can deal with two damage problems: damage characterized by a function of maximum strain attained in a loading path and damage that is strain-rate-dependent (i.e., the viscoelastic effect).

In the former, there is no damage accumulation, and the maximum strain energy function without damage,${\text{Max }}\tilde{\psi }\left( {{\mathbf{F}}\left( t \right)} \right),$ during a loading path can serve as the failure criterion. In the latter, however, the damage accumulation exists during a cyclic loading path, and the maximum effective strain energy function, $\int_{0}^{t} {\left| {{{d\tilde{\psi }\left( {{\mathbf{F}}\left( s \right)} \right)} \mathord{\left/ {\vphantom {{d\tilde{\psi }\left( {{\mathbf{F}}\left( s \right)} \right)} {ds}}} \right. \kern-0pt} {ds}}} \right|} ds,$ should be used as the damage criterion.

These two damage mechanisms [9, 10] were combined by Miehe [7] with two sets of damage variables in the continuum damage mechanics method shown above. Since the model includes the viscoelastic effect, which is beyond the scope of this review, it is not further discussed here.

### Damage Model for Abdominal Aortic Aneurysms

A damage model for abdominal aortic aneurysms (AAAs) was proposed [11] based on the above continuum damage mechanics method. AAAs are considered as a compressible, homogenous, and isotropic material without any fibers. The strain energy function with damage is written as:

14 $$ \psi \left( {\mathbf{C}} \right) = \tilde{\psi }_{vol} \left( J \right) + \left( {1 - d} \right)\tilde{\psi }\left( {{\bar{\mathbf{C}}}} \right) $$

where ${\mathbf{C}} = {\mathbf{F}}^{\mathbf{T}} {\mathbf{F}},\; J = \det \left( {\mathbf{F}} \right), \; \overline{\mathbf{C}} = J^{{ - {2 \mathord{\left/ {\vphantom {2 3}} \right. \kern-0pt} 3}}} {\mathbf{C}}, \; {\mathbf{F}} = \partial {\mathbf{x}} /\partial {\mathbf{X}},\;{\mathbf{x}}$ is the current configuration, **X** represents the reference configuration, and *d* is the damage variable, which can be calculated as:

15 $$ d\left( {\alpha \left( t \right)} \right) = a\left[ {1 - \exp \left( { - b\alpha \left( t \right)} \right)} \right] $$

where $\alpha \left( t \right) = \sqrt {2\tilde{\psi }\left( {{\mathbf{C}}\left( t \right)} \right)},$ and *a* and *b* are model constants. This equation is the same as Eq. (8). Note that AAA tissues demonstrate anisotropic biomechanical properties [12–14], obviously, this isotropic damage may not be justified.

The energy limiter method is another alternative for dealing with the damage effect in isotropic materials such as rubber or rubber-like materials [15]. For solids, the energy limiter for damage/failure/rupture is equivalent to the bond energy, which can be measured using the strain energy function. The following constitutive law for AAA was proposed by Volokh [16] based on an isotropic strain energy plus an energy limiter:

16 $$ \psi \left( {\mathbf{C}} \right) = \phi - \phi \exp \left\{ { - {{\left[ {c_{1} \left( {tr{\mathbf{C}} - 3} \right) + c_{2} \left( {tr{\mathbf{C}} - 3} \right)^{2} } \right]} \mathord{\left/ {\vphantom {{\left[ {c_{1} \left( {tr{\mathbf{C}} - 3} \right) + c_{2} \left( {tr{\mathbf{C}} - 3} \right)^{2} } \right]} \phi }} \right. \kern-0pt} \phi }} \right\} $$

where $\phi$ is the energy limiter, and *c*_1_ and *c*_2_ are material constants. These three model parameters need to be determined using uniaxial tension test data of AAAs. Note that there is no need to involve a material damage variable in this damage model, as described in Sect. 3.1.

## Damage Models for Anisotropic Soft Tissues

### Deterministic Damage Models

The artery wall is incompressible, nonlinear, and inhomogeneous, and exhibits hysteresis under a cyclic load. Its fibrous structure (i.e., collagen and elastin fibers) can be torn under a pressure higher than the physiological pressure. This micro-tearing is strain-related and contributes to the amount of damage. Like a rubber material, damage to the arterial wall is closely related to the maximum strain.

Under a steady load, the artery cannot be damaged until the maximum strain is achieved. Under a cyclic load, the loading and unloading stress–strain paths will remain unchanged until a previous maximum strain is exceeded. Such behavior is referred to as the Mullins or softening effect, which was originally used to describe rubber behavior.

In traditional methods, once the maximum von Mises stress or strain at a point in a material is beyond a criterion, the material is said to experience failure. However, such local failure does not lead to total failure in an artery. This means that traditional methods for predicting the failure of rubber materials may be unsuitable for arteries, and thus new methods are needed for predicting total failure in the arterial wall.

Arterial tissues are subject to the softening effect and have an S-shaped stress–stretch curve [17–20]. In [21], an anisotropic damage model was proposed to account for tensile and compressive damage based on continuum damage mechanics. This model has been applied to artery damage prediction [22]. However, fibers and the softening effect were excluded. It seems to be difficult to extend this model to tissues with fibers. Hence, this model is not further discussed here.

In the following paragraphs, we summarize a few kinds of fiber-based damage model. The important one is the artery biomechanical model proposed by Holzapfel et al. [23] updated with the damage effect.

The constitutive law is expressed by the strain energy function put forward by Holzapfel et al. [23] for arterial walls with an incompressible, homogenous matrix and two families of collagen fibers:

17 $$ \psi \left( {I_{1},I_{4},I_{6} } \right) = \frac{\mu }{2}\left( {I_{1} - 3} \right) + \frac{{k_{1} }}{{2k_{2} }}\sum\limits_{i = 4,6} {\left\{ {\exp \left[ {k_{2} \left( {I_{i} - 1} \right)^{2} } \right] - 1} \right\}} $$

where *I*_1_, *I*_4_, and *I*_6_ are the stretch invariants, and *I*_*i*_ = **f**_**i**_•(**Cf**_**i**_), *i* = 4, 6, where **f**_**i**_ is the orientation vector of each family of fibers.

To accommodate the softening effect in the matrix material, the neo-Hookean model of the first term in Eq. (17) is updated as [24]:

18 $$ \psi \left( {I_{1},\phi } \right) = \phi - \phi \exp \left\{ { - \frac{\mu }{2\phi }\left( {I_{1} - 3} \right)} \right\} $$

The last two terms are modified as:

19 $$ \psi \left( {I_{4},I_{6},\xi_{4},\xi_{6},n_{4},n_{6} } \right) = \frac{{k_{1} }}{{2k_{2} }}\sum\limits_{i = 4,6} {\left\{ {\exp \left[ {k_{2} \left( {I_{i} - 1} \right)^{2} } \right] - 1 - \frac{{k_{2} }}{{2n_{i} + 1}}\left( {\frac{{I_{i} - 1}}{{\xi_{i}^{2} - 1}}} \right)^{{2n_{i} + 1}} } \right\}} $$

where $$ \phi,$$$$ \xi_{i}, $$ and $$ n_{i} $$ are the damage parameters determined from experiments; the parameters $$ \mu, $$$$ k_{1}, $$ and $$ k_{2} $$ are model constants without damage. This model is applicable for each layer of arterial walls.

In [25], the damage model described by Eqs. (18) and (19) was modified using a strain energy limiter, sharpness factor, and upper incomplete gamma function as follows:

20 $$ \psi \left( {I_{1},\phi } \right) = \frac{\phi }{m}\left\{ {\Upgamma \left( {\frac{1}{m},0} \right) - \Upgamma \left( {\frac{1}{m},\left[ {\frac{{\frac{\mu }{2}\left( {I_{1} - 3} \right)}}{\phi }} \right]^{m} } \right)} \right\} $$

and

21 $$ \psi \left( {I_{4},I_{6},\phi_{4},\phi_{6},m_{4},m_{6} } \right) = \sum\limits_{i = 4,6} {\frac{{\phi_{i} }}{{m_{i} }}\left\{ {\Upgamma \left( {\frac{1}{{m_{i} }},0} \right) - \Upgamma \left( {\frac{1}{{m_{i} }},\left[ {\frac{{\frac{{k_{1} }}{{2k_{2} }}\left( {e^{{k_{2} \left( {I_{i} - 1} \right)^{2} }} - 1} \right)}}{{\phi_{i} }}} \right]^{{m_{i} }} } \right)} \right\}} $$

where the parameters $$ \phi,$$$$ \phi_{4}, $$ and $$ \phi_{6} $$ are the strain energy limiters for the matrix and two families of fibers, respectively, *m*, *m*_4_, and *m*_6_ are the sharpness factors for the matrix and two families of fibers, respectively, which can be determined from uniaxial or biaxial tensile test data, and Γ represents the upper incomplete gamma function, defined as $$ \Upgamma \left( {s,x} \right) = \int\limits_{x}^{\infty } {t^{s - 1} e^{ - t} dt} . $$

Similarly, a damage model for soft tissues with fibers was proposed by Ehret and Itskov [26] to account for the softening effect. The generalized poly-convex strain energy function for an isotropic matrix and anisotropic fibers to meet the material stability criteria was adopted. The initial stiffness of fibers was reduced gradually to fit the experimental stress–stretch curves under cyclic loadings, suggesting that damage occurs only in the fibers.

Based on the continuum damage mechanics model described in Sect. 2.2, a damage model for artery walls was proposed by Peña et al. [27] and Balzani et al. [28]. It is considered that damage occurs in two families of fibers only. As a result, the strain energy function of the matrix material does not need to be modified. Only the last two energy functions for the fibers are updated to the following form:

22 $$ \psi \left( {I_{4} ,I_{6} ,d_{4} ,d_{6} } \right) = \frac{{k_{1} }}{{2k_{2} }}\sum\limits_{i = 4,6} {\left\{ {\exp \left[ {k_{2} \left\langle {\left( {1 - d_{i} } \right)\left( {\kappa I_{1} + \left( {1 - 3\kappa } \right)I_{i} } \right) - 1} \right\rangle^{2} } \right] - 1} \right\}} $$

where *d*_*i*_ is the damage variable of fibers and *κ* is a constant associated with fiber orientation dispersion. Details can be found elsewhere [29]. This treatment of the damage effect is slightly different from that in Eq. (19).

Further, the following type of damage variable was applied to represent the damage process for the case where the maximum loading is fixed in a cyclic tension test:

23 $$ d_{i} \left( \beta \right) = d_{s} \left[ {1 - \exp \left( {\frac{{\ln \left( {1 - r_{s} } \right)}}{{\beta_{s} }}\beta } \right)} \right] $$

where $\beta = \int_{0}^{t} {d\tilde{\psi }},$ and *r*_*s*_ and *β*_*s*_ are model parameters, where *β*_*s*_ is the variable at *r*_*s*_ = 0.99, $r_{s} \in \left[ {0,1} \right] .$ The maximum damage variable *d*_*s*_ is determined using:

24 $$ d_{s} = d_{\infty } \left[ {1 - \exp \left( {\frac{{\ln \left( {1 - r_{\infty } } \right)}}{{\alpha_{\infty } }}\alpha } \right)} \right] $$

where *d*_∞_ denotes a predefined convergence limit for the overall damage value, $d_{\infty } \in \left[ {0,1} \right] ,$ and *α*_∞_ is the variable at *r*_∞_ = 0.99, $r_{\infty } \in \left[ {0,1} \right] .$ The maximum strain energy function is $\alpha \left( t \right) = {\text{Max }}\tilde{\psi }\left( {{\mathbf{F}}\left( t \right)} \right) .$ These two damage variables can be also found in [7].

In [27], based on discontinuous and continuous damage models [7], a damage model for the pig aorta under cyclic loads was proposed. The damage occurs in both the matrix and fibers. The damage variables are $d_{i} = d_{i} \left( {\alpha_{i} \left( t \right)} \right) + d_{i} \left( {\beta_{i} \left( t \right)} \right) ,$*i* = m, 4, 6 for the matrix and two families of fibers, respectively, where $\alpha_{i} \left( t \right) = {\text{Max}}\sqrt {2\tilde{\psi }_{i} \left( {{\mathbf{F}}\left( t \right)} \right)}$ and $\beta_{i} \left( t \right) = \int_{0}^{t} {\left| {{{d\tilde{\psi }_{i} \left( {{\mathbf{F}}\left( s \right)} \right)} \mathord{\left/ {\vphantom {{d\tilde{\psi }_{i} \left( {{\mathbf{F}}\left( s \right)} \right)} {ds}}} \right. \kern-0pt} {ds}}} \right|} ds .$ The strain energy function with damage for the matrix and two families of fibers is written as:

25 $$ \psi = \psi_{vol} \left( J \right) + \left( {1 - d_{m} } \right)\tilde{\psi }_{m} + \left( {1 - d_{4} } \right)\tilde{\psi }_{4} \left( {I_{4} } \right) + \left( {1 - d_{6} } \right)\tilde{\psi }_{6} \left( {I_{6} } \right) $$

where $$ \tilde{\psi }_{m} , $$$$ \tilde{\psi }_{4} , $$ and $$ \tilde{\psi }_{6} $$ are the strain energy functions of the matrix and two families of fibers without damage, respectively. The damage criterion for the discontinuous damage process is:

26 $$ \phi_{i} \left( {{\mathbf{F}}\left( i \right),\zeta_{i} } \right) = \sqrt {2\tilde{\psi }_{i} \left( {{\mathbf{F}}\left( t \right)} \right)} - \alpha_{i} \left( t \right) = \zeta_{i} \left( t \right) - \alpha_{i} \left( t \right) \le 0 $$

The damage variable evolution equation updated by Peña et al. [27] is expressed as:

27 $$d_{i} \left( {\zeta_{i} } \right) = \left\{ {\begin{array}{lll} 0
& \quad  \zeta_{\text{i}} < \zeta_{i}^{\hbox{min}}  \\ \frac{1}{2}\left[ 1+ \frac{2\xi_{i} \Uplambda_{i} \exp \left( 2\xi_{i} \left[2\Uplambda_{i} - 1 \right] \right) - 1}{2\xi_{i} \Uplambda_{i} \exp\left( 2\xi_{i} \left[ 2\Uplambda_{i} \right] \right) + 1} \right] & \quad  \zeta_{\text{i}} \in \left[\zeta_{i}^{\hbox{min}},\zeta_{i}^{\hbox{max}} \right]\\ 1 & \quad  \zeta_{\text{i}} >\zeta_{i}^{\hbox{max} } \end{array} } \right. $$

where the variable $\Uplambda_{i} = {{\left( {\zeta_{i} - \zeta_{i}^{\hbox{min} } } \right)} \mathord{\left/ {\vphantom {{\left( {\zeta_{i} - \zeta_{i}^{\hbox{min} } } \right)} {\left( {\zeta_{i}^{\hbox{max} } - \zeta_{i}^{\hbox{min} } } \right)}}} \right. \kern-0pt} {\left( {\zeta_{i}^{\hbox{max} } - \zeta_{i}^{\hbox{min} } } \right)}} ,$ and $\zeta_{i}^{\hbox{min} }$ and $\zeta_{i}^{\hbox{max} }$ are the strain energy function values for damage occurrence and total failure, respectively. The model parameter $\xi_{i}$  > 0. The continuous damage variable $d_{i} \left( \beta \right)$ has the following form:

28 $$ d_{i} \left( \beta \right) = d_{i\infty } \left[ {1 - \exp \left( { - \frac{\beta }{{\gamma_{i} }}} \right)} \right] $$

where $d_{i\infty }$ is the maximum possible continuous damage in the matrix and fibers, $d_{i\infty } \in \left[ {0,1} \right] ,$ and $\gamma_{i}$ is the damage saturation parameter. This model requires ten experimental damage parameters and five constitutive law constants. Although the discontinuous and continuous damage models have good agreement with experiments, the plastic effect is not presented in the model in [27].

In [30], a damage model was proposed for arterial tissue that includes softening and plastic phenomena. The damage in both the matrix and collagen fibers was taken into account by introducing two damage variables. Note that the strain energy function is an exponential function rather than neo-Hookean type. The strain energy function is Eq. (17). The two damage variables yield Eq. (28). This model includes the softening and plastic effects, and thus has excellent agreement with observations.

Damage models for cerebral arterial tissue have been developed [31, 32]. It was considered that the damage occurs in elastin fibers only. The isotropic model for the elastin fibers is in terms of the following strain energy function:

29 $$ \psi_{elastin} \left( {I_{1} } \right) = \frac{{k_{1} }}{{2k_{2} }}\left\{ {\exp \left[ {k_{2} \left( {I_{1} - 3} \right)} \right] - 1} \right\} $$

The strain energy function for fibers can be that proposed by Holzapfel et al. [23] or Gasser et al. [29]. The strain energy function with elastin damage is written as:

30 $$ \psi \left( {I_{1} ,I_{4} ,I_{6} ,d} \right) = \left( {1 - d} \right)\tilde{\psi }_{elsastin} \left( {I_{1} } \right) + \tilde{\psi }_{fibre} \left( {I_{4} ,I_{6} } \right) $$

where the damage variable *d* can be determined using two approaches, one of which is:

31 $$ d\left( {I_{1} } \right) = \left\{ {\begin{array}{*{20}l} {0 \quad I_{1} - 3 < \alpha_{f} } \hfill \\ {1 \quad I_{1} - 3 \ge \alpha_{f}} \hfill \\ \end{array} } \right. $$

where *α*_*f*_ is the experimental damage threshold for elastin fibers in cerebral artery; the other damage function for cyclic loadings is expressed as:

32 $$ \left\{ \begin{aligned} d &= 1 - \left( {1 - d_{1} }\right)\left( {1 - d_{2} } \right)\left( {1 - d_{3} } \right) \hfill\\ d_{i} &= \left\{ \begin{array}{ll} 0 &\alpha_{\text{i}} < \alpha_{si}  \hfill \\  \frac{1 - \exp \left[{c_{i} \left( 1 - {{\alpha_{i}} \mathord{\left/ {\vphantom{{\alpha_{i} } {\alpha_{fi} }}} \right. \kern-0pt} {\alpha_{fi} }}\right)} \right]}{1 - \exp \left[ {c_{i} \left( {1 - {{\alpha_{i} }\mathord{\left/ {\vphantom {{\alpha_{i} } {\alpha_{si} }}} \right.\kern-0pt} {\alpha_{si} }}} \right)}\right]} & \alpha_{i} \in [\alpha_{si} ,\alpha_{fi} ) \hfill \\ 1 & \alpha_{i} \ge \alpha_{fi} \hfill \\ \end{array}  \right. \end{aligned} \right. $$

where *i* = 1, 2, 3, which indicate three damage mechanisms of elastin fibers in the cerebral artery, namely the damage due to maximum strain $$ \alpha_{1} , $$$$ \alpha_{1} \left( t \right) = {\text{Max}}\sqrt {2\tilde{\psi }\left( {I_{1} } \right)} , $$ the damage due to accumulated equivalent strain $$ \alpha_{2} , $$$$ \alpha_{2} = \int_{0}^{t} {\left| {{{d\sqrt {2\psi \left( {I_{1} } \right)} } \mathord{\left/ {\vphantom {{d\sqrt {2\psi \left( {I_{1} } \right)} } {dt}}} \right. \kern-0pt} {dt}}} \right|} dt , $$ and the damage due to the haemodynamic shearing effect on the arterial wall $$ \alpha_{3} , $$$$ \alpha_{3} = f\left( \textit{shears\,tress} \right) , $$ where $$ \alpha_{si} $$ and $$ \alpha_{fi} $$ are the damage start and complete failure thresholds for these damage mechanisms, which need to be determined from experiments. $$ c_{i} $$ is another model parameter.

The work for isotropic rubber-like materials [3] described in Sect. 2.1 was extended to materials with organized fibers, for example, for the artery wall by Peña and Doblaré [33], Weisbecker et al. [19], and Pierce et al. [34]. Like Eq. (1), the strain energy function of a material with damage is written as:

33 $$ \psi = \psi_{vol} \left( J \right) + \sum\limits_{{i = m,_{{f_{1} ,f_{2} }} }} {[\eta_{i} \tilde{\psi }_{i} } + \phi_{i} (\eta_{i} )] $$

where $\psi \left( J \right)$ is the strain energy function for material volume change, which has nothing to do with damage. The damage function $\phi_{i} \left( {\eta_{i} } \right)$ is given by:

34 $$ - \frac{{d\phi_{i} }}{{d\eta_{i} }} = \alpha_{i} {\mathbf{erf}}^{ - 1} \left( {\beta_{i} \left( {\eta_{i} - 1} \right)} \right) + \tilde{\psi }_{i}^{0} $$

with the boundary condition $$ \phi_{i} \left( 1 \right) = 0 . $$$$ \tilde{\psi }_{i}^{0} $$ is the strain energy function at the primary loading path. The damage variable is like that in Eq. (4):

35 $$ \eta_{i} = 1 - \frac{1}{{\beta_{i} }}{\mathbf{erf}}\left( {\frac{{\tilde{\psi }_{i}^{0} - \tilde{\psi }_{i} }}{{\alpha_{i} + \gamma_{i} \tilde{\psi }_{i}^{0} }}} \right) $$

where *α*_*i*_, *β*_*i*_ and *γ*_*i*_ are positive material damage constants, which need to be determined from experiments. The minimum value of *η*_*i*_ is determined as:

36 $$ \eta_{i}^{0} = 1 - \frac{1}{{\beta_{i} }}{\mathbf{erf}}\left( {\frac{{\tilde{\psi }_{i}^{0} }}{{\alpha_{i} + \gamma_{i} \tilde{\psi }_{i}^{0} }}} \right) $$

where $\eta_{i} \in \left[ {\eta_{i}^{0} ,1} \right] .$ This damage model was applied to identify the material parameters of the porcine carotid artery subjected to a uniaxial cyclic test in the longitudinal and circumferential directions [35]. The strain energy function for fibers proposed by Holzapfel et al. [36] was used. The 13 model parameters were determined using an optimization method against a series of stress–strain experimental data under various cyclic loadings.

In [37], a damage model was developed for vaginal tissue that is composed of a homogenous matrix and one family of fibers. The model is based on the assumption that damage exists in both the matrix and fibers. Introducing two damage variables, *d*_*m*_ and *d*_*f*_, the damage strain energy function is written as:

37 $$ \psi = \psi_{vol} \left( J \right) + \left( {1 - d_{m} } \right)\tilde{\psi }_{m} + \left( {1 - d_{f} } \right)\tilde{\psi }_{f} $$

where $\tilde{\psi }_{m}$ and $\tilde{\psi }_{f}$ are the strain energy functions of the matrix and fibers without damage, respectively. The damage criterion resembles Eq. (26):

38 $$ \left\{ \begin{array}{l} \sqrt {2\tilde{\psi }_{i} \left( t\right)} - \alpha_{i} \left( t \right) \le 0 \hfill \\ \alpha_{i}\left( t \right) = {\text{Max}}\sqrt {2\tilde{\psi }_{i} \left( t\right)} \hfill \\ \end{array} \right.\quad i = m,f $$

The damage variables *d*_*m*_ and *d*_*f*_ can be determined using the following empirical correlation:

39 $$ d_{i} = \left\{ \begin{array}{ll} 0 & \alpha_{\text{i}} \left( t\right) < \alpha_{i}^{\hbox{min} } \\ \xi^{2} \left[ {1 - \beta_{i}\left( {\xi^{2} - 1} \right)} \right] & \alpha_{i} \left( t \right)\in \left[ {\alpha_{i}^{\hbox{min} },\alpha_{i}^{\hbox{max} } }\right] \hfill \\ 1& \alpha_{\text{i}} \left( t \right) >\alpha_{i}^{\hbox{max}} \hfill \\ \end{array} \right.\quad i = m,f$$

where $$ \xi = {{\left[ {\alpha_{i} \left( i \right) - \alpha_{i}^{\hbox{min} } } \right]} \mathord{\left/ {\vphantom {{\left[ {\alpha_{i} \left( i \right) - \alpha_{i}^{\hbox{min} } } \right]} {\left[ {\alpha_{i}^{\hbox{max} } - \alpha_{i}^{\hbox{min} } } \right]}}} \right. \kern-0pt} {\left[ {\alpha_{i}^{\hbox{max} } - \alpha_{i}^{\hbox{min} } } \right]}} , $$$$ \alpha_{i}^{\hbox{min} } $$ and $$ \alpha_{i}^{\hbox{max} } $$ are the damage variables indicating the damage start and complete failure, respectively, for the matrix and fibers, and *β*_*i*_ is a material parameter, $$ \beta_{i} \in \left[ { - 1,1} \right] . $$

The strain energy function for the matrix material is the well-known neo-Hookean type $\tilde{\psi }_{m} = c\left( {I_{1} - 3} \right) .$ However, the strain energy function for the one family of fibers is slightly complicated:

40 $$ \tilde{\psi }_{f} = \left\{\begin{array}{ll} 0 & I_{4}< I_{40} \hfill \\ \frac{{k_{1} }}{{k_{2} }}\left\{ {\exp \left[{k_{2} \left( {I_{4} - I_{40} } \right)} \right] - k_{2} \left({I_{4} - I_{40} } \right) - 1} \right\}& I_{4} > I_{40} ,I_{4}< I_{4ref} \hfill \\ 2k_{3} \sqrt {I_{4} } + k_{4} \ln \left({I_{4} } \right) + k_{5} & I_{4} > I_{4ref} \end{array} \right.$$

where $I_{4ref}$ is the stretch squared beyond which collagen fibers start to become straightened, $I_{40}$ is the stretch squared at which the collagen fibers begin to engage a loading. The model parameters, *c*, *k*_1_ through *k*_5_, $\alpha_{i}^{\hbox{min} } ,\; \alpha_{i}^{\hbox{max} } , \; \beta_{i} ,\;I_{40},$ and *I*_4*ref*_ need to be determined from a series of experimental stress–strain curves. This model has been used to identify the parameters of vaginal tissue [37] and the rectus and sheath [38].

The continuum damage mechanics models for matrix material and fibers in [39] are very similar to those in Eqs. (37)–(39). The only difference is in the damage variable formula, and thus these modes are not further discussed below.

In [40], another type of strain energy function for fibers and the continuum mechanics damage variable *d* were used in the damage model. Since this model just combines previous work, it is not discussed here.

The damage model proposed in [28] was updated in [41] by introducing the proteoglycan bridge damage of collagen fibrils. The bridge damage is modeled with statistical processes and related to the damage variable *d*. The statistical proteoglycan orientation (beta distribution) and bridge internal length (Gaussian distribution) contribute to the bridge damage process.

In the damage models mentioned above, a standard continuum damage formulation and damage variable are used, because the damage effect in a soft tissue is a natural result of cross-section reduction of a specimen of the tissue. This means that the material strength degradation is in a local sense. Such a local effect can result in an ill-posed problem and increased mesh refinement, especially for soft tissues usually subjected to a significant deformation [42]. To overcome this geometrically non-linear effect, a non-local gradient damage formulation has been presented [42]. In this formulation, two extra energy functions were added into the strain energy function, proposed in [29], to include the damage effect. The first extra energy function is the scalar product of the gradient of a non-local damage variable and a scalar field variable with respect to three coordinates in the current configuration; the second extra energy function is the penalty function of the squared diffidence between the scalar field variable and the usual damage variable. Based on the principle of minimum potential energy, a second-order partial differential equation of the non-local damage variable was established. The usual damage variable was the source term of that partial differential equation. Like in other damage models, the stress in the fibers is degraded by making use of the ordinary exponential damage function of the usual damage variable. Since an additional non-local damage variable has to be solved during damage simulation, this approach may be time-consuming.

### Probabilistic Damage Models of Fibers

A damage model was proposed by Chu and Blatz [43] for cat mesentery. The stress–stretch curves of biaxial test specimens of cat mesentery exhibit hysteresis. This is mainly due the cumulative microdamage mechanism that occurs in the collagen fibers. To model this effect, it is assumed that the stretch in a region is statistically distributed among fibers and that the stresses are statistically distributed among the remaining unbroken fibers. Based on the Ogden-type strain energy function in Eq. (2), the curves were fitted by adjusting the property constants in the strain energy function and the parameters in the probability density function of stress. This may be the very first damage model for soft tissue.

In [39, 44, 45], a stochastic damage model for fibers was proposed and a comparison of the predictive capability between the model and the continuum damage mechanics model was made, with similar results obtained. In the stochastic damage model, the damage model for the matrix material is an ordinary one, like those mentioned above; but the fiber damage model is slightly different. However, the strain energy function of individual fibers in [44] is quite different from that in [39, 45]. The proper choice of function is not clear. In the following descriptions, the specific form of the strain energy function for a fiber is thus omitted.

In the loading-free state, a collagen fiber is wavy. With increasing loading, it starts to become straight until it fails. The total strain energy function of all fibers expressed by Rodriguez et al. [45] is:

41 $$ \tilde{\psi }_{f} \left( \lambda \right) = \left\{\begin{array}{ll} 0& \lambda < 1 \hfill \\ \int_{0}^{\lambda}\int_{{l_{\hbox{max} } }}^{l} {\sigma_{f} \left( {\xi ,x}\right)}p\left( x \right)dxd\xi & \lambda \ge 1 \hfill \\ \end{array} \right. $$

where *σ*_*f*_ is the stress in a fiber, $\sigma_{f} = {{\partial \tilde{\psi }_{1} } \mathord{/ {\vphantom {{\partial \tilde{\psi }_{1} } {\partial \lambda }}}  \kern-0pt} {\partial \lambda }} , \; \tilde{\psi }_{1}$ is the strain energy function of a fiber that takes a form based on eight-chain model proposed by Arruda and Boyce [46] for rubber elastic materials, and *p*(*x*) is a beta probability density function with parameters *m* and *n* to account for stress variation among fibers:

42 $$ p\left( x \right) = \frac{1}{{l_{\lim } - l_{0} }}\frac{{\Upgamma \left( {m + n} \right)}}{\Upgamma \left( m \right)\Upgamma \left( n \right)}\left( {\frac{{x - l_{0} }}{{l_{\lim } - l_{0} }}} \right)^{n - 1} \left( {1 - \frac{{x - l_{0} }}{{l_{\lim } - l_{0} }}} \right)^{m - 1} \, x \in \left[ {l_{0} ,l_{\lim } } \right] $$

where *l*_0_ is the reference length of fibers, $l_{\hbox{max} } = \exp \left[ {\left( {{x \mathord{\left/ {\vphantom {x \delta }} \right. \kern-0pt} \delta }} \right)^{\theta } } \right]l_{0} \lambda_{\hbox{max} } ,\;  \lambda_{\hbox{max} }$ is the maximum stretch of fibers without failure over the time history in a test (if $\lambda \ge \lambda_{\hbox{max} }$ then a failure starts to occur), *δ* and *θ* are the model parameters, and *l*_lim_ is the failure stretch limit (when a stretch reaches this value, the fiber breaks completely).

A ligament is a soft tissue that connects two bones in a joint. Ligaments stabilize joints and guide their motion when a tensile load is applied [47]. Ligaments are composed of collagens (approximately 75 % of the dry weight), proteoglycans (<1 %), elastin, other proteins (glycoproteins, such as actin, laminin, and integrins), and some water, which may be responsible for cellular function and viscoelastic behavior [47, 48].

The ligament microstructure comprises collagen bundles aligned along the long axis of the ligament and exhibits a wavy or crimp pattern along the length, which allows the ligament to elongate without sustaining damage after a load is applied.

Ligaments demonstrate passive nonlinear anisotropic biomechanical behavior only. At a low loading, they are relatively compliant because of crimped collagen fibers and the viscoelastic effect; at a high loading, however, they are much stiffer because fibers are recruited and straightened [47]. Ligaments are quite often damaged in traumatic joint injuries. The damage includes partial ligament failure or complete ligament tear [47]. The ligament stress–stretch curve has an S-shape.

Based on the recruitment models of collagen fibers in [49–51], see Appendix 1 for details, a probabilistic damage model for ligaments was developed by Liao and Belkoff [52]. To derive the damage model, several assumptions were made: (1) only the fibers in the ligament respond to a loading; (2) the interaction between the matrix and fibers and that among fibers are ignored; (3) the viscoelastic effect of a ligament is not taken into account; (4) the initial shape of fibers is wavy in the stress-free state and has a Gaussian distribution; (5) all the fibers are linearly elastic and have the same elastic modulus and limit strain; (6) fibers experience brittle failure and they fail in the same sequence in which they are recruited. The number of fibers recruited is given by the following expression:

43 $$ dn = \frac{n}{{s\sqrt {2\pi } }}\exp \left[ { - \frac{1}{2}\left( {\frac{{x - \overline{x} }}{s}} \right)^{2} } \right] $$

where *n* is the total number of fibers. The total force generated by all the fibers when they are subjected to a stretch is given by the equation:

44 $$ F\left( \lambda \right) = \frac{{nA_{i} E_{i} }}{{s\sqrt {2\pi } }}\int_{1}^{\lambda } {\left( {\frac{\lambda - x}{x}} \right)} \exp \left[ { - \frac{1}{2}\left( {\frac{{x - \overline{x} }}{s}} \right)^{2} } \right]dx $$

where *A*_*i*_ and *E*_*i*_ are the cross-section and Young’s modulus of a fiber, respectively. Then, the mean stress in the fibers is:

45 $$ \sigma \left( \lambda \right) = \frac{F\left( \lambda \right)}{{nA_{i} }} = \frac{{E_{i} }}{{s\sqrt {2\pi } }}\int_{1}^{\lambda } {\left( {\frac{\lambda - x}{x}} \right)} \exp \left[ { - \frac{1}{2}\left( {\frac{{x - \overline{x} }}{s}} \right)^{2} } \right]dx $$

At a breaking strain *ε*_lim_, or a breaking stretch $\lambda_{\lim } = 1 + \varepsilon_{\lim},$ some straightened fibers fail. The stress due to the contributions of fibers stretched beyond $\lambda /\lambda_{\lim }$ is:

46 $$ \sigma \left( {{\lambda \mathord{\left/ {\vphantom {\lambda {\lambda_{\lim } }}} \right. \kern-0pt} {\lambda_{\lim } }} \ge 1} \right) = \frac{{E_{i} }}{{s\sqrt {2\pi } }}\int_{1}^{{{\lambda \mathord{\left/ {\vphantom {\lambda {\lambda_{\lim } }}} \right. \kern-0pt} {\lambda_{\lim } }}}} {\left( {\frac{\lambda - x}{x}} \right)} \exp \left[ { - \frac{1}{2}\left( {\frac{{x - \overline{x} }}{s}} \right)^{2} } \right]dx $$

The resultant stress after failure should be equal to $\sigma \left( \lambda \right) - \sigma \left( {{\lambda \mathord{\left/ {\vphantom {\lambda {\lambda_{\lim } }}} \right. \kern-0pt} {\lambda_{\lim } }} \ge 1} \right) ,$ and thus we have the stress after failure as:

47 $$ \sigma \left( \lambda \right) = \frac{{E_{i} }}{{s\sqrt {2\pi } }}\int_{{{\lambda \mathord{\left/ {\vphantom {\lambda {\lambda_{\lim } }}} \right. \kern-0pt} {\lambda_{\lim } }}}}^{\lambda } {\left( {\frac{\lambda - x}{x}} \right)} \exp \left[ { - \frac{1}{2}\left( {\frac{{x - \overline{x} }}{s}} \right)^{2} } \right]dx \, \quad \lambda \ge \lambda_{\lim }. $$

Before failure, i.e., $\lambda \le \lambda_{\lim } ,$ the stress can be estimated using Eq. (47). The parameters *E*_*i*_, $\overline{x} ,$*s*, and *λ*_lim_ need to be determined from experimental stress–stretch curves.

Another idea for modeling ligament rupture failure is that the damage of a ligament is a gradual reduction of its fiber stiffness at a randomly distributed stretch threshold rather than a constant limit strain or stretch. This type of damage model was proposed in [53, 54] based on a stretch threshold described by the Weibull distribution, which is frequently used to describe the random yield strength or fatigue life of a material [55].

In the model, the collagen fibers carry the load applied on a ligament, and the elastin contribution is neglected. The interaction between fibers and the matrix material is not taken into account. The collagen fibers are linearly elastic and their orientation is parallel to the loading direction. The fibers are wavy in the stress-free sate, but they become straightened with increasing loading until damage occurs. The fiber recruitment effect is ignored, however, and the collagen straightening stretch *λ*_*s*_ and stretch threshold for damage, *λ*_*f*_, are considered as statistical variables specified by a Weibull distribution.

The Weibull probability distribution function for the collagen straightening stretch *λ*_*s*_ at which fibers are straight and ready to engage a load is:

48 $$ P_{s} \left( \lambda_{s},\alpha_{s} ,\beta_{s} ,\gamma_{s}\right) = \left\{ \begin{array}{ll} 0 & for \; \lambda_{\text{s}}< \gamma_{s} \hfill \\ 1 - \exp \left[  - \left(\frac{\lambda_{s} - \gamma_{s}}{\beta_{s}} \right)^{{\alpha_{s}}}  \right] & for \; \lambda_{\text{s}} < \gamma_{s} \hfill \\ \end{array} \right. $$

where the parameter $\gamma_{s} = 1,$ and the other two positive parameters, *α*_*s*_ and *β*_*s*_, are determined based on the microstructure of a specimen in the stress-free state or from the stress–stretch curve. The inverse function of Eq. (48) is:

49 $$ \lambda_{s} (P_{s} ;\alpha_{s} ,\beta_{s} ,\gamma_{s} ) = \gamma_{s} + \beta_{s} \left[ { - \ln \left( {1 - P_{s} } \right)} \right]^{{{1 \mathord{\left/ {\vphantom {1 {\alpha_{s} }}} \right. \kern-0pt} {\alpha_{s} }}}} $$

Let us assume that there are *n* fibers in a specimen. The initial length of the *i*-th fiber in the specimen, $\lambda_{s}^{(i)} ,$ can be calculated as:

50 $$ \lambda_{s}^{(i)} (P_{s}^{(i)} ;\alpha_{s} ,\beta_{s} ,\gamma_{s} ) = \gamma_{s} + \beta_{s} \left[ { - \ln \left( {1 - P_{s}^{(i)} } \right)} \right]^{{{1 \mathord{\left/ {\vphantom {1 {\alpha_{s} }}} \right. \kern-0pt} {\alpha_{s} }}}} $$

where $P_{s}^{(i)}$ is a random number between 0 and 1.

It is assumed that fiber damage occurs in *m* fibers once a limit stretch is exceeded, which is a random variable described by a Weibull function. This means that damage consists of a series of sub-failures. Thus, the *j*-th sub-failure of the *i*-th fiber is expressed by the following Weibull distribution:

51 $$ \lambda_{d}^{(j)} (P_{d}^{(j)} ;\alpha_{d} ,\beta_{d} ,\gamma_{d} ) = \gamma_{d} + \beta_{d} \left[ { - \ln \left( {1 - P_{d}^{(j)} } \right)} \right]^{{{1 \mathord{\left/ {\vphantom {1 {\alpha_{d} }}} \right. \kern-0pt} {\alpha_{d} }}}} $$

Similarly, $P_{d}^{(j)}$ is a random number between 0 and 1; $\gamma_{d}$ is the mean limit stretch and can be determined easily from experiments. For example, for rat medial collateral ligaments, $\gamma_{d} = 1.0514$ [56]. The remaining positive parameters *α*_*d*_ and *β*_*d*_ need to be optimized from experimental data.

All fibers have the same Young’s modulus, *k*. When damage occurs, the fiber modulus will degrade gradually until a complete tear. It is assumed that the damage process conforms to an exponential law [54]. The damage variable *d* is related to the stress by the follow expression:

52 $$ \sigma^{(i)} \left( {k,d,\lambda^{(i)} ,\lambda_{s}^{(i)},\lambda_{d}^{(j)} } \right) = \left\{ \begin{array}{ll} 0 &\lambda^{(i)} \le \lambda_{s}^{(i)}  \hfill \\ k\left({\lambda^{(i)} - \lambda_{s}^{(i)} } \right) & \lambda^{\left( i\right)} \in \left( {\lambda_{s}^{(i)} ,\lambda_{d}^{\left( j\right)} } \right) \hfill \\ d^{j} k\left( \lambda^{(i)} -\lambda_{d}^{(j)}  \right) & \lambda^{\left( i \right)} \ge \lambda_{d}^{(j)} \hfill \\ \end{array}  \right. $$

where the damage variable d ∈ (0,1) and modulus *k* need to be determined from experimental stress–stretch curves.

Finally, the stress in the specimen is defined as the mean of the stresses of *n* fibers, expressed by:

53 $$ \sigma \left( \lambda \right) = \frac{1}{n}\sum\limits_{i = 1}^{n} {\sigma^{(i)} \left( {k,d,\lambda^{(i)} ,\lambda_{s}^{(i)} ,\lambda_{d}^{(j)} } \right)} $$

The model above is subject to the set of parameters $$ \left\{ {k,d,\alpha_{s} ,\beta_{s} ,\gamma_{s} ,\alpha_{d} ,\beta_{d} ,\gamma_{d,} n,m} \right\} . $$ Since $$ \gamma_{s} = 1, $$$$ \gamma_{d} = 1.0514, $$*n* = 10^5^, and *m* = 10^2^ [54], the model requires six parameters: $$ \left\{ {k,d,\alpha_{s} ,\beta_{s} ,\alpha_{d} ,\beta_{d} } \right\} . $$

The early version of the model above is also interesting [53]. In that model, there is no damage variable and it is supposed that once a limit stretch is exceeded, a fiber fails rather than breaking step by step. Moreover, it is assumed that the limit stretch itself is a random variable described by the Weibull function:

54 $$ \lambda_{f}^{(i)} = \gamma_{f} + \beta_{f} \left[ { - \ln \left( {1 - P_{f}^{\left( i \right)} } \right)} \right]^{{{1 \mathord{\left/ {\vphantom {1 {\alpha_{f} }}} \right. \kern-0pt} {\alpha_{f} }}}} $$

where $\lambda_{f}^{\left( i \right)}$ is the failure stretch of the *i*-th fiber. Accordingly, the stress in that fiber is given by:

55 $$ \sigma^{(i)} \left( {k,\lambda^{(i)} ,\lambda_{s}^{(i)},\lambda_{f}^{(j)} } \right) = \left\{\begin{array}{ll} 0 & \lambda^{(i)} \le \lambda_{s}^{(i)}  \hfill \\ k\left({\lambda^{(i)} - \lambda_{s}^{(i)} } \right) & \lambda^{\left( i\right)} \in \left(\lambda_{s}^{(i)} ,\lambda_{f}^{\left( j \right)}\right) \hfill \\ 0 & \lambda^{\left( i \right)} \ge \lambda_{f}^{(j)} \hfill \\ \end{array}  \right. $$

This model is subject to five parameters $\left\{ {k,\alpha_{s} ,\beta_{s} ,\alpha_{f} ,\beta_{f} } \right\}$ only and its performance is very satisfactory.

The tendon is a connecting tissue between muscles and bone and can be damaged after being excessively stretched. The tendon is mainly composed of collagen fibers, some proteoglycans, and fluid, and thus it can respond to a loading passively. The mechanical properties of tendons were investigated in vitro by Schechtman and Bader [57, 58]. A typical stress–strain relationship obtained from a simple tensile test [57] has an S-shape.

It is considered that a human tendon is a collagen-fiber-reinforced composite nonlinear material with a uniform matrix that may be compressible [59]. The fibers are wavy in the load-free state; if a load is applied in the physiological force direction, they are stretched but do not generate passive tension until straightened.

The biomechanical interaction between the matrix material and fibers as well as the viscous effect (strain-rate-dependent feature) are not taken into account. In [59], the damage model is based on the following strain energy function:

56 $$ \psi ({\mathbf{C}}) = U(J) + \psi_{m} (\bar{I}_{1} ,\bar{I}_{2} ) + \psi_{f} (I_{4} ) $$

where **C** is the right Cauchy-Green tensor, $$ {\mathbf{C}} = {\mathbf{F}}^{\mathbf{T}} {\mathbf{F}} , $$$$ \bar{I}_{1} $$ and $$ \bar{I}_{2} $$ are the principal invariants associated with the iso-volumetric components of the right Cauchy-green tensor, $$ {\bar{\mathbf{C}}} = J^{{ - {2 \mathord{\left/ {\vphantom {2 3}} \right. \kern-0pt} 3}}} {\mathbf{C}} , $$ and $$ I_{4} $$ is the squared stretch along the fiber orientation:

57 $$ I_{4} = a_{0} \cdot {\mathbf{C}}a_{0} = \lambda^{2} $$

where *a*_0_ is the vector of fiber orientation and *λ* is the stretch.

The matrix material volume term *U*(*J*) is defined as:

58 $$ U(J) = K(J - 1)^{2} $$

where *K* is the modulus of the material (*K* = 1000 MPa) [59]. The iso-volumetric term of the matrix material is expressed as:

59 $$ \psi_{m} (\bar{I}_{1} ,\bar{I}_{2} ) = c_{1} (\bar{I}_{1} - 3) + c_{2} (\bar{I}_{2} - 3) $$

where the property constants $c_{1}$ and $c_{2}$ are the initial tangent shear modulus of the matrix material and need to be determined from experimental data. The strain energy function for fiber response is written as [59]:

60 $$ \tilde{\psi }_{f} (I_{4} ) = \frac{{k_{1} }}{{k_{2} }}\left[ {e^{{k_{2} (I_{4} - 1)}} - k_{2} (I_{4} - 1) - 1} \right] $$

where *k*_2_ is the parameter associated with the initial crimp of the fiber and *k*_1_ is the initial stiffness of the fiber. Equation (60) requires $I_{4} \ge 1 .$

For a tendon, damage occurs to fibers only (the matrix material is not damaged). The fiber damage function is associated with the last term $\psi_{f} (I_{4} )$ in Eq. (56). The stain energy function with the fiber damage effect takes the following form [59]:

61 $$ \psi ({\mathbf{C}},d) = U(J) + \psi_{m} (\bar{I}_{1} ,\bar{I}_{2} ) + g(d)\tilde{\psi }_{f} (I_{4} ) $$

The fiber damage function is related to the fiber stretch and expressed as [59]:

62 $$ g(d) = \frac{{1 - e^{{\beta (\lambda^{4} - \lambda_{\lim }^{4} )}} }}{{1 - e^{{\beta (\lambda_{{^{0} }}^{4} - \lambda_{\lim }^{4} )}} }} $$

where *β* is a parameter related to the initial wavy state of the fiber, *β* < 0, *λ*_0_ is the maximum stretch without fiber damage, and *λ*_lim_ is the fiber stretch at which all fibers are broken. If *λ* is between *λ*_0_ and *λ*_lim_, then the fiber damage function, *g*(*d*), will be engaged in Eq. (62); otherwise, *g*(*d*) = 1.

The damage variable *d* is defined as the ratio of the number of damaged fibers, *n*_*b*_, to the total number of fibers, *n*, in a test sample of tissue:

63 $$ d = \frac{{n_{b} (\lambda )}}{n} $$

Since the crimped state of fibers varies with the sample, each fiber should start to be damaged at its own critical stretch. It is assumed that such an effect is in accordance with the Gaussian distribution [49]. The number of damaged fibers, $$ n_{b} , $$ is expressed as:

64 $$ n_{b} (\lambda ) = \frac{n}{{s\sqrt {2\pi } }}\int_{{\lambda_{0} }}^{\lambda } {\text{e}^{{ - \frac{{(x - \bar{\lambda })^{2} }}{{2s^{2} }}}} dx} $$

where $\bar{\lambda }$ and *s* are the mean value and standard deviation of the critical stretch for damage of an individual fiber, respectively. They are determined from experimental data. Eventually, the damage variable *d* takes the following form:

65 $$ d(\lambda ) = \frac{1}{{s\sqrt {2\pi } }}\int_{{\lambda_{0} }}^{\lambda } {\text{e}^{{ - \frac{{(x - \bar{\lambda })^{2} }}{{2s^{2} }}}} dx} $$

It is related to the stretch during damage by a linear equation, i.e.:

66 $$ \lambda = \lambda_{0} + (\lambda_{1} - \lambda_{0} )d $$

If a series of experimental stretch data and the values of $$ \lambda_{0} $$ and $$ \lambda_{1} $$ are available, Eq. (66) can be used to calculate a series of $$ d $$; then, $$ \bar{\lambda } $$ and $$ s $$ can be determined by fitting the scattered $$ d - \lambda $$ plot with Eq. (65). Eventually, the relationship among $$ g(d) , $$$$ d , $$ and $$ \lambda $$ can be obtained. The parameters $$ c_{1} , $$$$ c_{2} , $$ and $$ k_{1} $$ are obtained from experimental stress–strain data. The parameters $$ k_{2} $$ and $$ \beta $$ are associated with the wavy state of fibers under the load-free condition. For a cyclic loading, *k*_2_ = 10 and *β* = −2.8 (fibers are very wavy); for a steadily increasing loading, *k*_2_ = 25 and *β* = −1.0 (fibers are less wavy) [59].

Theoretically, *I*_4_ in Eq. (56) should be $\bar{I}_{4} ,$ which is associated with the iso-volumetric components of the right Cauchy-Green tensor, ${\bar{\mathbf{C}}} ,$ i.e., $\overline{I}_{4} = a_{0} \cdot \overline{\mathbf{C}} a_{0} = \lambda^{2}$ [23]. Therefore, the justification of the damage models expressed with Eq. (61) needs to be clarified in the future.

### Microstructure-Based Damage Model of Fibers

Studies have investigated constitutive models of vascular tissue and its damage modeling [60, 61] based on the microstructure of collagen fibrils in vascular wall tissue. The major contents of vascular tissue are elastin, collagen, and proteoglycans; in particular, collagen fibers play a very important role in determining the biomechanical properties of the tissue. It was shown that a series of proteoglycan (PG) bridges can be formed to generate force as soon as collagen fibrils become straightened (*λ*_*st*_ = 1) by stretch. The first Piola–Kirchhoff stress generated in a fibril is given as the following equation by employing a triangle probability density function [61]:

67 $$ T\left(\lambda \right) = \left\{ \begin{array}{ll} 0 & \lambda\in \left(0,\lambda_{\hbox{min}} \right] \hfill \\ \frac{2k}{{3\Delta \lambda^{2} }}\left(\lambda -\lambda_{\hbox{min}}\right)^{3} & \lambda \in \left(\lambda_{\hbox{min}} ,\overline{\lambda} \right] \hfill \\ k\left[\lambda - \tfrac{{2\left( {\lambda - \lambda_{\hbox{max} } }\right)^{3} }}{{3\Delta \lambda^{2} }} - \overline{\lambda}\right]&\lambda \in \left(\overline{\lambda } ,\lambda_{\hbox{max}} \right]\hfill \\ k\left( \lambda - \overline{\lambda} \right) & \lambda \in \left(\lambda_{\hbox{max} } ,\infty  \right) \hfill \\ \end{array} \right. $$

where $$ \Delta \lambda = \lambda_{\hbox{max} } - \lambda_{\hbox{min} } , $$$$ \overline{\lambda } = 0.5\left( {\lambda_{\hbox{min} } + \lambda_{\hbox{max} } } \right) , $$ and $$ k $$ is the stiffness of a collagen fibril. The Cauchy stress of a collagen fibril is $$ \sigma \left( \lambda \right) = \lambda T\left( \lambda \right) $$; usually, $$ \lambda_{st} = \lambda_{\hbox{min} } = 1, $$ and *λ*_max_ = 2. The Cauchy stress in the fibers of tissue is expressed as:

68 $$ \sigma_{fibre} = \int\limits_{V} {\rho \left( {\text{N}} \right)} \sigma \left( {\lambda \left( {\text{N}} \right)} \right)dev\left( {{\text{n}} \otimes {\text{n}}} \right)dV $$

where $\rho \left( {\text{N}} \right)$ is the orientation density function of fiber bundles, ${\text{n}}$ is the direction vector of a fibril, and $V$ is the total volume of a vascular tissue. The total Cauchy stress in the tissue is calculated as:

69 $$ \sigma = \sigma_{vol} + \sigma_{nH} + \sigma_{fibre} $$

where $\sigma_{vol}$ and $\sigma_{nH}$ are attributed to the volumetric energy function $\psi_{vol} = K\left( {J - 1} \right)^{2} ,$ and the neo-Hookean strain energy function. Such a treatment for the stress in fibers involves the collagen recruitment concept.

The damage is assumed to occur in fibers only due to a load [60]. A damage variable is involved in the second Piola–Kirchhoff stress of a collagen fibril:

70 $$ S = \left( {1 - d} \right)\tilde{S} = \left( {1 - d} \right)k\left( {{\lambda \mathord{\left/ {\vphantom {\lambda {\lambda_{st} - 1}}} \right. \kern-0pt} {\lambda_{st} - 1}}} \right) $$

where the damage variable $$ d = 1 - \exp \left[ { - a\left( {{{\lambda_{st} } \mathord{\left/ {\vphantom {{\lambda_{st} } {\lambda_{st0} }}} \right. \kern-0pt} {\lambda_{st0} }}} \right)^{2} } \right] , $$$$ a $$ is a model parameter, $$ \lambda_{st0} $$ is the stretch of a fibril becoming straightened initially, and $$ \lambda_{st} $$ is the stretch of a fibril becoming straightened later. Because of plastic deformation, the relation $$ \lambda_{st} \le \lambda_{st0} $$ is kept. The damage criterion for fibers is written as [60]:

71 $$ \left\{ \begin{array}{ll} \tilde{S} < Y_{0} & {\text{ elastic deformation, no damage}} \hfill \\ \tilde{S} = Y_{0} + H & {\text{ plastic deformation, damage}} \hfill \\ \tilde{S} > Y_{0} + H & {\text{ complete damage}} \hfill \\ \end{array} \right. $$

where $\tilde{S}$ is the second Piola–Kirchhoff stress without damage, $Y_{0}$ is the elastic limit, $H$ represents the hardening effect due to the slowly (viscous) sliding mechanism in proteoglycan bridges, $H = \eta {{d\lambda_{st} } \mathord{\left/ {\vphantom {{d\lambda_{st} } {dt}}} \right. \kern-0pt} {dt}} ,$ and $\eta$ is an experimental coefficient. $\lambda_{st}$ increases with time $t .$ The Cauchy stress in a fibril is obtained as $\sigma = J^{ - 1} S\lambda^{2} .$ Equations (67) to (71) represent the damage model for collagen fibers in vascular tissue based on their microstructure proposed by Gasser [61]. Strictly speaking, this damage model is probabilistic.

This damage model relies on the irreversible sliding damage of PG bridges across collagen fibrils. It was shown that PG bridges exist in cartilage and tendons [62–64]. Whether they exist in vascular tissue needs to be confirmed by microscopic observation. In addition, the sliding damage effect or plastic deformation needs to be clarified at microscopic experimental level.

## Discussion and Challenges

In the reviewed damage models, there are a few parameters that are determined using a series of experimental data for a steady or cyclic load. Usually, they are determined mathematically by means of optimization or the least squares method under the condition that the error in the stress–stretch curve between measured and predicted values is the minimum.

The experimental data can be from uniaxial or biaxial tensile or shear tests or inflation measurements of a segment of organ or soft tissue. For organs, FEA is used to get the Cauchy stresses for a certain load, which is a time-consuming optimization procedure.

### Experimental Data Curve-Fitting

In this section, a few important cases are given to illustrate the feasibility of damage models. In order to show the discontinuous damage effect, the results predicted by the damage model proposed in [27] are demonstrated. The arterial tissue of animals exhibits softening behavior during a uniaxial tensile test under cyclic loads [27]. This behavior has been investigated theoretically based on the damage model in Eqs. (22)–(28). The model parameters are *μ* = 0.072294 MPa, *d*_*m*_ = *d*_4_ = *d*_6_ = 0.00006, $$ \zeta_{i}^{\hbox{min} } $$ = 0.08, $$ \zeta_{i}^{\hbox{max} } = 0.41, $$$$ \xi_{i} = 0.61, $$$$ d_{i\infty } = 0.507, $$ and $$ \gamma_{i} = 15.0 $$ for the matrix, and *k*_1_ = 0.000582 MPa, *k*_2_ = 3.675678, $$ \zeta_{i}^{\hbox{min} } = 0.01, $$$$ \zeta_{i}^{\hbox{max} } = 0.465, $$$$ \xi_{i} = 1.06, $$*d*_4_ = *d*_6_ = 0.00006, $$ d_{i\infty } = 0.507, $$ and $$ \gamma_{i} = 2.55 $$ for the fibers. A combination of discontinuous and continuous damage models can predict the softening/damage effect better than can either model alone.

**Fig. 3:**
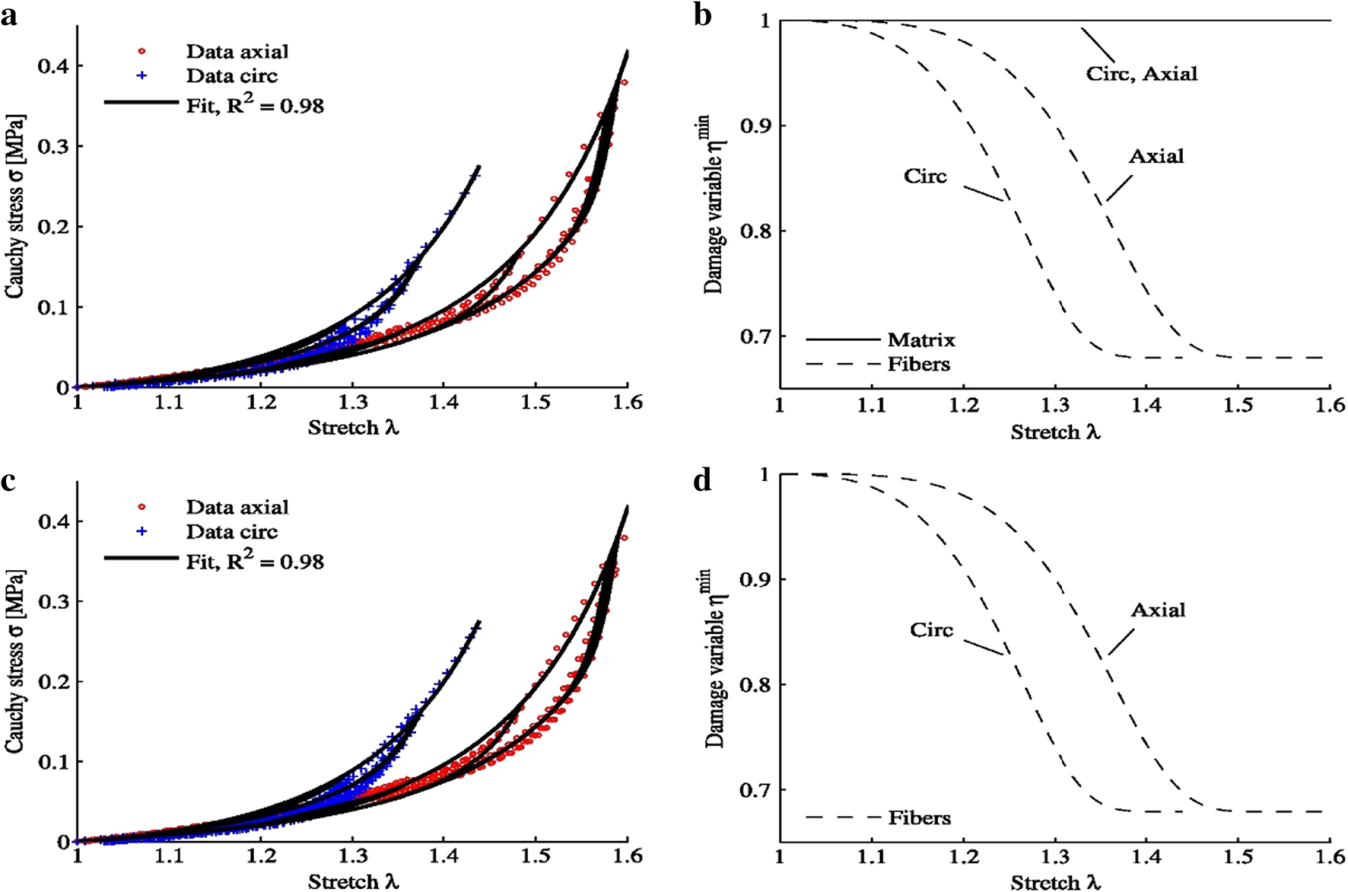
Experimental and predicted Cauchy stress-stretch curves and damage variable variation under cyclic loads. **a**, **b** Damage of collagen fibers, **c**, **d** damage of media, from [19]

The isotropic damage model in [3] has been extended to anisotropic cases. This extended damage model has potential applications in biomedical engineering [19, 33, 34]. Figure 3 shows the experimental and predicted Cauchy stress–stretch curves and damage variable variation under cyclic loads for arteries given by Weisbecker et al. [19]. The experimental data are fitted very well.

**Fig. 4:**
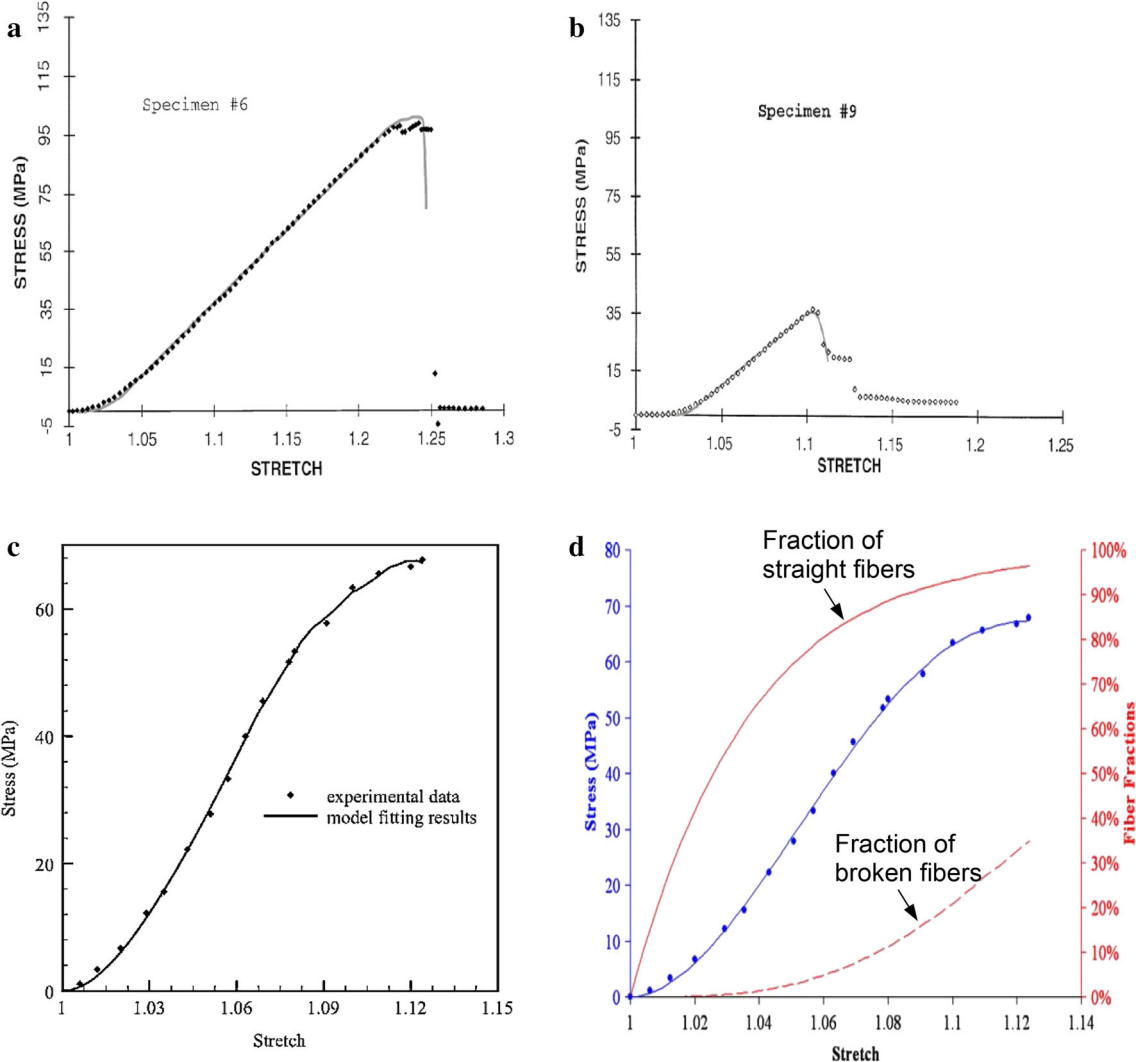
Comparison of experimental stress-stretch data of ligaments and their prediction made by damage models proposed respectively by **a**, **b** Liao and Belkoff [52], **c** Guo and De Vita [54], and **d** De Vita and Slaughter [53]. *Symbols* are experimental data and *lines* are model prediction results

Figure 4 shows the predicted stress–stretch curve of the ligaments harvested from two groups of rabbits based on the probabilistic damage model proposed by Liao and Belkoff [52]. It can be seen that the abrupt failure behavior of ligaments is captured very well. Figure 4 also shows the performance of models proposed by De Vita and Slaughter [53] and Guo and De Vita [54] for freshly harvested rat medial collateral ligaments. Although both models produce an S-shape curve, the stress–stretch curve obtained by Guo and De Vita [54] is not smooth enough.

**Fig. 5:**
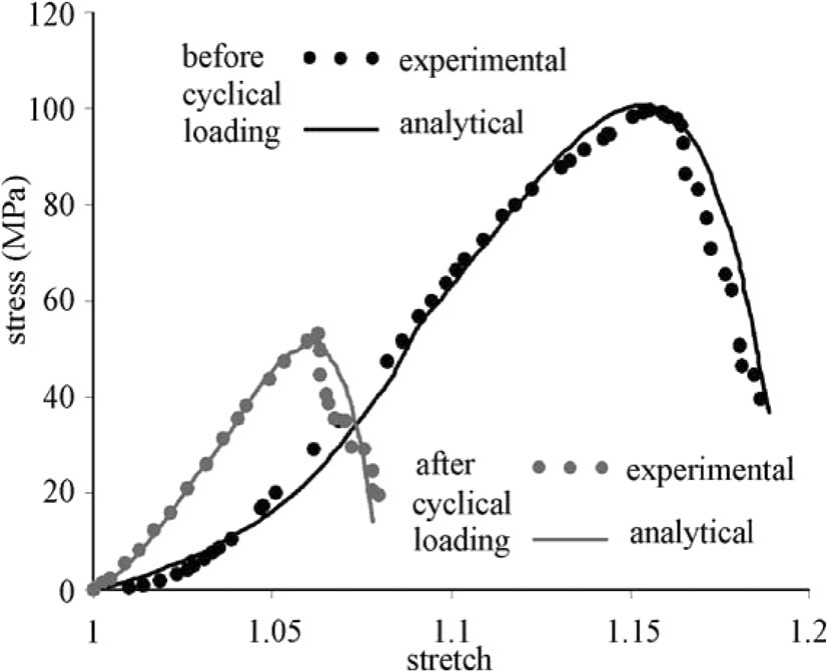
Stress–stretch curves for human tendon before and after cyclic loading, from [59]

The damage model proposed by Natali et al. [59] was used to represent experimental data of human tendons before and after cyclic loadings. Two curves are shown in Fig. 5, where the model parameters in Eqs. (56)–(66) are *K* = 1000 MPa, *c*_1_ = 1.0 MPa, *c*_2_ = 2.0 MPa, *k*_1_ = 4.0 MPa, *k*_2_ = 10, *λ*_0_ = 1.09, and *λ*_1_ = 1.195 before cyclic loading, and *k*_2_ = 25, *λ*_0_ = 1.02, and *λ*_lim_ = 1.08 after cyclic loading. The S-shape curve is well predicted by the model; however, the curve is not as sharp as the experimental data.

Based on the comparisons above, it can be concluded that existing damage models can represent experimental stress–stretch data precisely.

### Computational Effort

The damage model proposed by Volokh [24, 25] is based on the strain energy function for the arterial wall proposed by Holzapfel et al. [23]. The softening effect is handled using the softening parameter $\phi ,$*ξ*_4_(=*ξ*_6_), and *n*_4_(=*n*_6_) or strain energy limiters and sharpness factors. Therefore, a cyclic loading is unnecessary. Hence, this damage model can be readily applied to the analysis of simple tensile test results of soft tissue. Moreover, this model can be easily handled with MATLAB code for simple structures, such as a tube.

For damage models with damage variables, experimental stress–stretch curves of soft tissue are required for determining the damage parameters. Usually, this kind of damage model is so complex that a FORTRAN UMAT subroutine is needed for ABAQUS, ANSYS, or other FEA packages to perform a FEA in them. If so, the first-order derivatives of the Cauchy stresses with respect to stretch components and damage variables are desirable. Since these derivatives cannot be expressed analytically, a numerical increment formulation is required.

In the damage models for ligaments and tendons, the experimental stress–stretch curves can be well predicted by employing the fiber recruitment effect and probabilistic strain failure limit. The limitations in these models are that damage exists in fibers only and that the fibers are linearly elastic. To remove these drawbacks, proper strain energy functions for the matrix and fibers should be developed and the damage mechanisms should be introduced into the matrix and fibers as well. Moreover, probabilistic damage models require extremely long computational time and their application to complex structures may not be easy.

### Application of Damage Models

The ultimate objective of generating soft tissue damage models is to apply them to disease diagnosis, surgery, surgeon training procedures, and the design and fabrication of artificial soft tissue. Even though many damage models have been available for soft tissues, their applications in biomedical engineering seem limited.

**Fig. 6:**
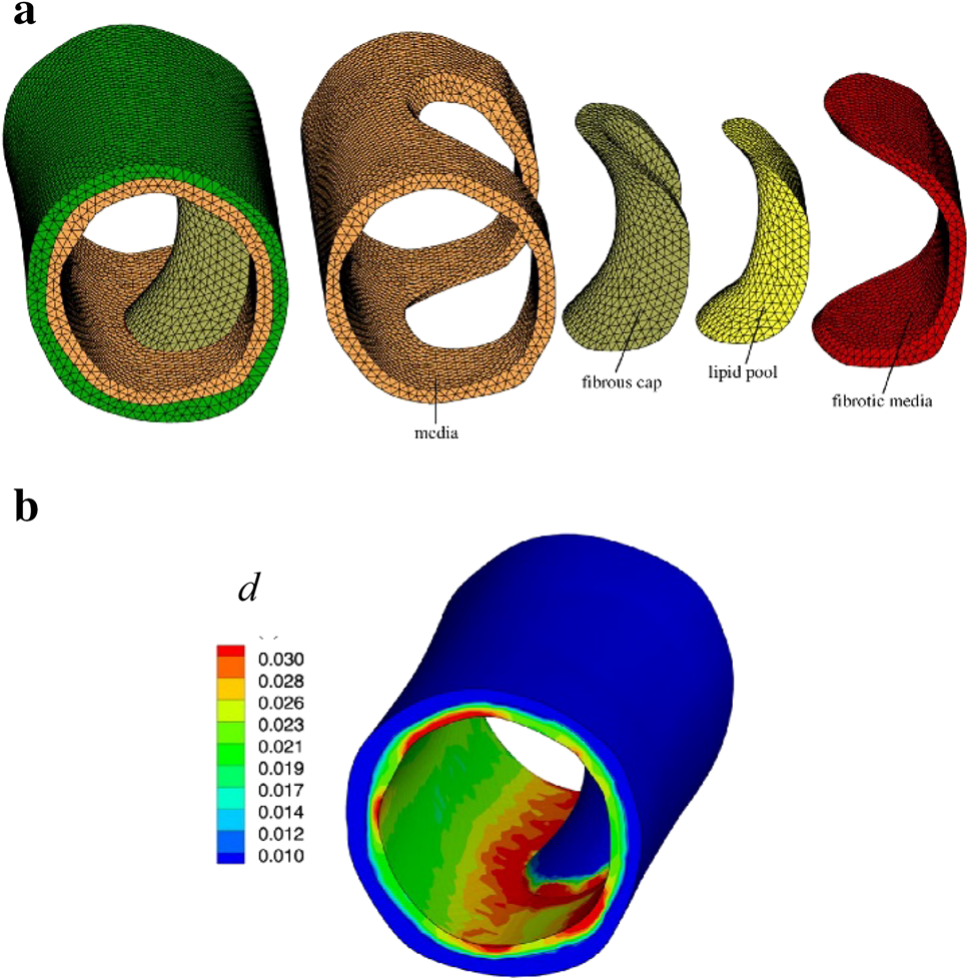
Damage variable distribution in human arterial wall under 80 kPa internal blood pressure. **a** 3D diseased arterial model and **b** damage variable distribution on wall, from [41]

Figure 6 shows the damage variable distribution on a human arterial media inner surface presented by Schmidt et al. [41] based on their own damage model with parameters extracted from circumferential and longitudinal uniaxial tests for human carotid artery media. It can be observed that the media experiences a serious damage effect around the fibrous cap.

The strength, von Mises stress, rupture potential index, and damage variable distributions on 10 patient-specific AAA walls under various blood pressures based on a damage model were reported by Marini et al. [11]. The damage developed in the areas with a high von Mises stress and a large rupture potential index. Even though the damage variable is correlated to AAA rupture slightly more poorly than to the von Mises stress, the damage variable still provides a useful link between a mechanical stimulus and the response of an AAA wall.

The applications of damage models in biomechanical engineering are exciting and convincing. Hence, more trials should be conducted in the future.

### Connection Between Damage and Fracture

Damage is closely related to fracture or crack extension/propagation in a soft tissue [4]. However, existing damage models are based on continuum damage mechanics and are macrostructure-based. The model parameters are usually obtained by fitting stress–stretch curves without any information about cracks. For soft tissues, a link between damage variables and crack propagation has not been established.

For fiber-reinforced soft tissues, a sub-failure or complete failure is driven by crack propagation in brittle failure or toughening in ductile failure inside a material. A visualization study of crack development or toughening during a simple tensile test needs to be conducted for soft tissues [65]. For a long-term objective, the crack characteristics or toughening behavior should be linked to the damage time-history profile and the damage behavior of soft tissues. For the visualization of cracking in soft tissue, damage patterns such as matrix cracking, fiber bridging, fiber rupture, fiber pull-out, and matrix/fiber de-bounding should be considered [66].

**Fig. 7:**
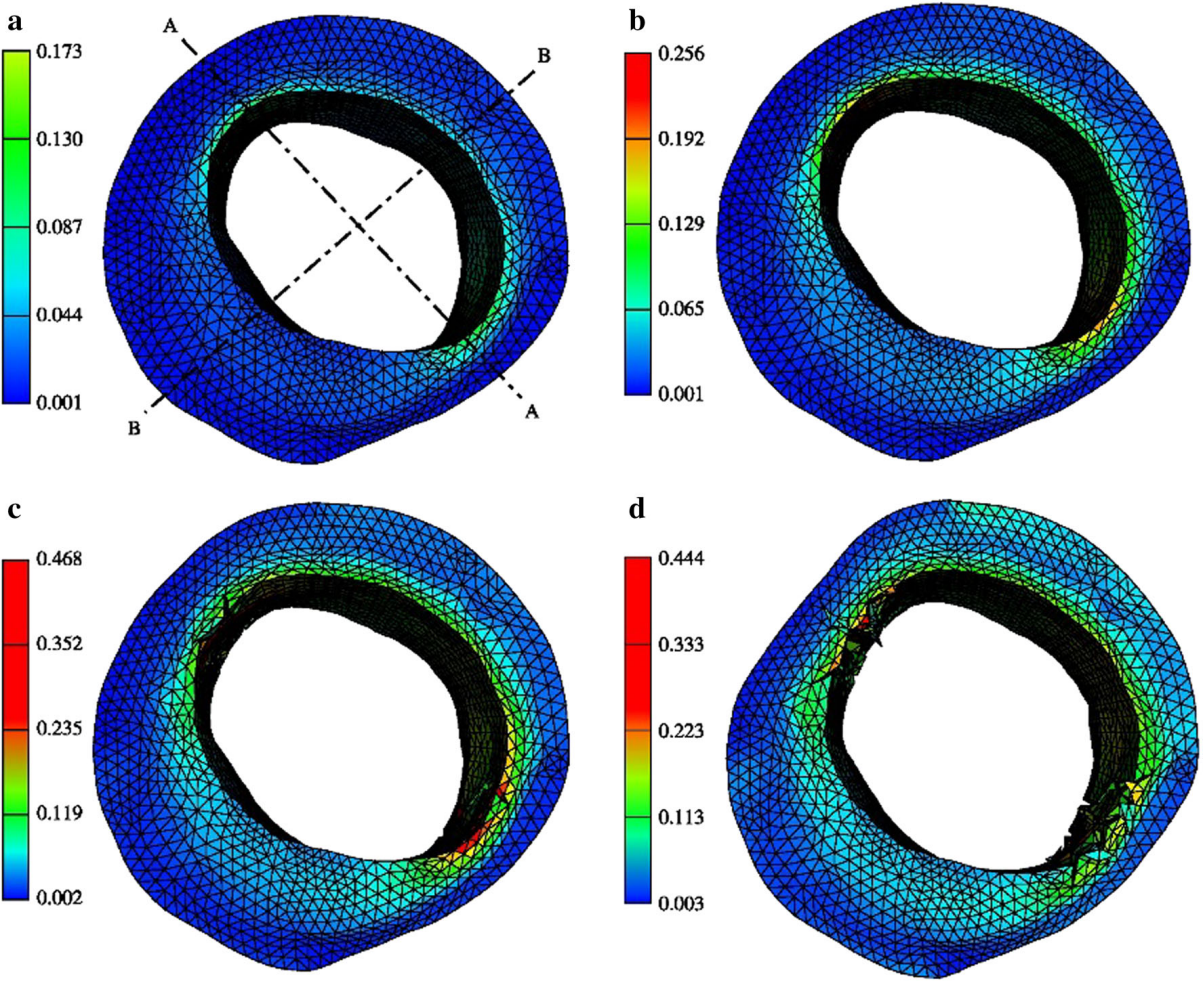
Crack generation and development in arterial wall under increasing inner blood pressure. **a** 100, **b** 120, **c** 180, and **d** 260 mmHg, from [69]

Numerical simulation of fracture propagation in soft tissue is equally important. Cohesive-zone law models are considered effective tools for tracking macro-crack propagation in a solid material under time-dependent loadings [67, 68]. As shown in Fig. 7, from Ferrara, Pandofi [69], with the cohesive-zone law model, crack generation and development in the arterial wall can be identified very clearly. With increasing inner blood pressure, more cracks are generated, and the existing cracks open widely and propagate deeply in the tissue.

**Fig. 8:**
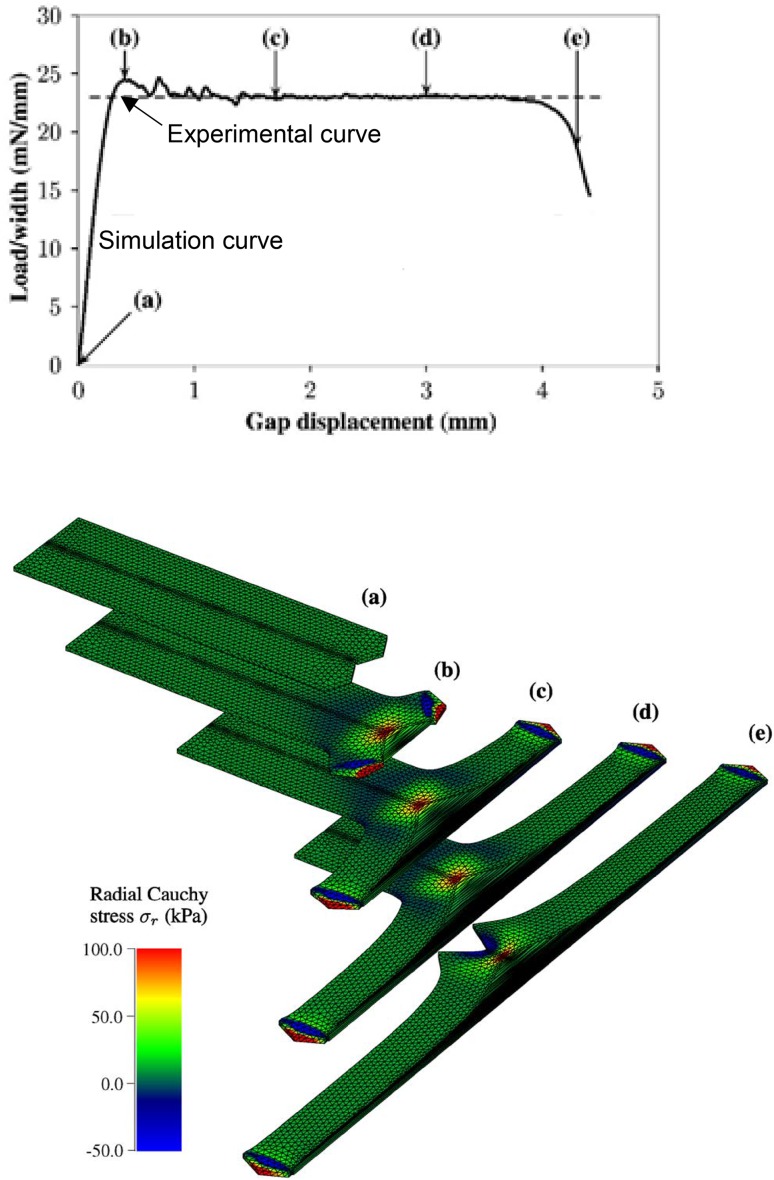
Predicted dissection process of 2D human aortic media obtained using isotropic cohesive model in [70], images from [70]

Another interesting study is the propagation of arterial dissection, which is frequently performed in clinical practice and can be caused by traffic accidents. A three-dimensional (3D) isotropic cohesive model was proposed by Gasser and Holzapfel [70] based on cohesive potential for human aortic media. The model involves cohesive tensile strength, two non-negative model parameters, and a damage variable that is the magnitude of the opening gap displacement of a crack. The model parameters are determined from the load-gap displacement curve obtained in a media dissection experiment. The radial Cauchy stress distributions predicted by the model during a dissection process of a two-dimensional (2D) human aortic media strip are shown in Fig. 8. This model can potentially be applied to the dissection of a 3D human aortic artery.

The material point method (MPM), a numerical method, can potentially be applied to track the cracks in soft tissues and characterize the fracture failure behavior of the tissue. MPM has advantages over traditional FEA methods since it can adapt to complex geometry, large deformation, and fragmentations that may occur in the fracture failure of soft tissue [71, 72].

### Other Issues

The viscoelastic effect may not be significant in damage to soft tissue [73]. However, a study showed that viscoelasticity is important for the damage modeling of elastic and viscoelastic materials [74]. Nevertheless, this problem needs to be clarified in detail. A method for estimating the dissipated energy via viscoelasticity in soft tissue has been proposed and compared with other methods [75].

It has been shown that discontinuous and continuous damage mechanisms are equally important for softening effect prediction in soft tissues [27, 28]. Nevertheless, more experimental evidence is required.

Soft tissue damage is relevant to tissue histological change and inflammation of cells. A well-defined relationship between the histological change and the inflammation of cells is unavailable. For smooth muscle, however, active stress is dominant but is not considered in any damage model.

It has been indicated that the soft tissue of left and right ventricles of animals also exhibits the softening effect under a cyclic loading [76, 77]. The softening effect with quite substantially plastic deformation was found for mouse skin [78] and ovine infrarenal vena cava tissue [79]. However, no damage model for myocardium or skin has been proposed.

Very soft tissues, such as those of the brain, liver, kidneys, and even skin exhibit a strong viscoelastic property and transversely isotropic behavior [73, 80–82]. Damage modeling for this kind of soft tissue is very important for automatic surgical tools and robots as well as surgeon training systems. When a surgical tool and robot is gasping a soft tissue with its gasper, the edges of the gasper can result in tissue injury because of stress concentration. It is assumed that once the peak stress is beyond a stress threshold, injury or damage can occur [2]. However, a proper in vivo stress threshold has not been reported. Damage models based on a strain energy function are necessary for very soft tissues at the moment.

## Conclusion

A series of state-of-the-art damage models for soft tissues in animals and humans, especially those that do not consider the viscoelastic effect, was reviewed. The damage of fiber-reinforced soft tissues can be treated by using updated strain energy functions with the softening effect or by including the fiber recruitment effect with damage variables. Fiber damage can be handled by employing a strain or stretch failure criterion with a probability distribution function. Existing damage models can produce stress–stretch curves that are in very good agreement with observations, but they are less applied in healthcare, surgery, and biomedical engineering. Further, the interaction between the matrix and fibers is ignored. To develop micro-level structure damage models, microstructure visualization is necessary during the damage process in soft tissue. Crack generation and propagation in soft tissues need to be measured and simulated with suitable numerical methods. The application of damage models in biomedical engineering and clinical practice should be extended. Damage models for very soft tissues (e.g., liver, brain, kidneys, and skin) are unavailable. The viscoelasticity of soft tissue needs to be rechecked and should be considered in damage models more properly. Active stress should be taken into account in damage models for smooth muscle.

## Figures and Tables

**Fig. 9:**
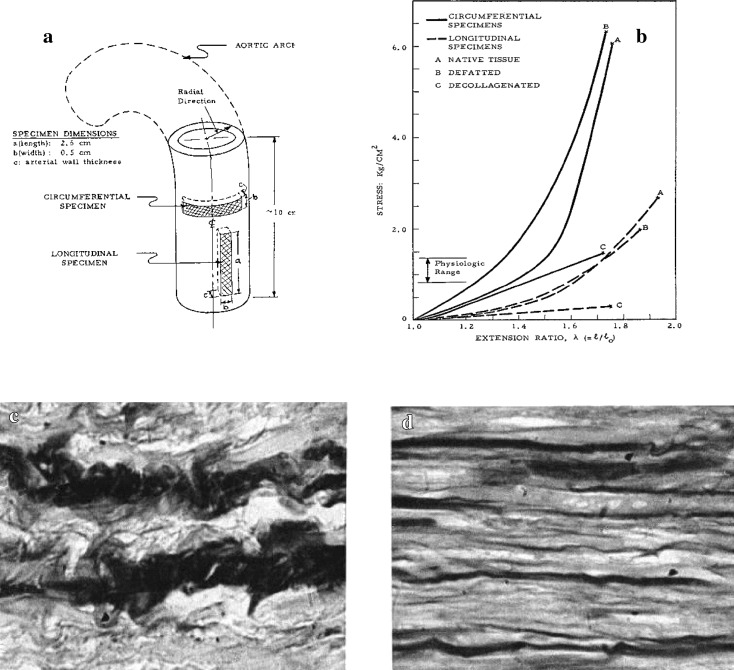
Two kinds of tensile specimen taken from different orientations and typical stress–extension measured, and pictures of a 5 μm section from a relaxed and stretched circumferential specimens in the ab plane, **a** specimen, **b** stress–extension curve, **c** relaxed state, **d** stretched by 1.5, aniline blue stain showing collagen fibrils, magnification ×1000, the figures are adapted from [49]

**Fig. 10:**
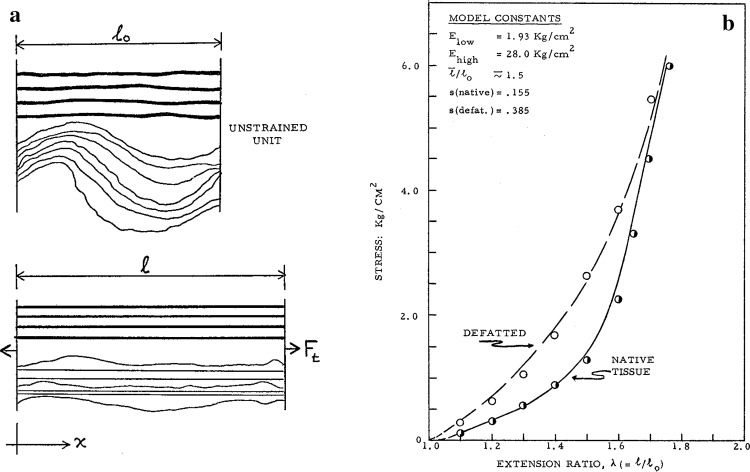
**a** Sketch of mechanical unit showing elastin fibers (*thick lines*) and collagen fibrils (*thin lines*) in parallel and **b** comparison of stress–extension curves predicted with model in Eq. (77) with experimental data, figures adapted from [49]

## Appendix 1: Recruitment Constitutive Models for Collagen Fibers

### Initial Recruitment Constitutive Model

The initial recruitment constitutive model is based on a series of uniaxial tests of a bovine upper descending aorta. In [49], the tensile test specimens were taken from a portion of the upper descending aorta of a 6-month-old bovine calf in the circumferential and longitudinal directions (see Fig. 9a). There are three kinds of specimen: native tissue that includes collagen fibers and lipids; defatted tissue; and tissue without collagen. The uniaxial simple test stress–extension curves of these tissue samples are shown in Fig. 9b. The response to a load in both directions is anisotropic. The circumferential response is stiffer than the longitudinal one for the native specimen. There are two distinct nearly linear deformation curves, from *λ* = 1.1 to 1.4 and *λ* = 1.6 to failure (curve A). Thus, two Young’s moduli can be defined: one for low stretch and one for high stretch.

After the lipids (fat) were removed, the sample lost some of its nonlinear behavior, giving the curve a large initial stiffness and a steadily increasing Young’s modulus (curve B). The sample without fat and collagen was basically a pure elastic material, exhibiting a strictly linear relation in the whole deformation region in both directions (curve C).

The structural anisotropy of collagen and elastic networks are illustrated in Fig. 9c and d. In the relaxed (stress-free) state, the collagen appears as diffuse, folded, micro-fibril bundles (see Fig. 9c). The elastin is somewhat wavy as well. In the tension state, Fig. 9d, the majority of the collagen fibrils become straightened, showing continuous bundles in the stress direction.

This histological evidence is consistent with the macroscopic stress–stretch (extension) observations in Fig. 9b. At low stretches (*λ* ≤ 1.4), the elastin fibers mainly carry the load, and nearly a pure linear stress–stretch curve is exhibited. At intermediate stretches (1.4 < *λ* ≤ 1.6), the collagen is straightened progressively, resulting in a sharp rise in Young’s modulus. Beyond 1.6, all the collagen has been straightened and bears the load, showing the highest stiffness. Wavy collagen becoming progressive straightened is referred to as collagen recruitment.

Based on histological observations in the load-free and load-engaged states, it was assumed that elastin fibers and collagen fibril bundles are arranged in parallel in a unit taken for a specimen, shown schematically in Fig. 10a. The initial (load-free state) length is *l*_0_ for the elastin fibers, which is equal to the initial length of the unit. In the load-free state, each collagen fibril has its own length *l*_*i*_, which is distributed with a specific probability around a mean value $\bar{l}$ under a standard deviation *s*. After a tensile force *F*_*t*_ is applied on both ends of the unit, the fibril length is extended to *l* from *l*_0_. This force is balanced by a retractive force generated in the elastin fibers and collagen fibril bundles:

72 $$ F_{t} = F_{elastin} + F_{collagen} = n_{e} E_{e} (l - l_{0} ) + E_{c} \sum\limits_{j = 1}^{n} {(l - l_{i,j} )} $$

where *E*_*e*_ and *E*_*c*_ are spring constants for the elastin and collagen, respectively, and *n*_*e*_ is the number of elastin fibers in the unit, which generate the retractive force. *n* is the number of collagen fibrils that start to generate a retractive force, that is, it is the number of collagen fibrils whose length is longer than the individual initial length *l*_*i*_. The relationship between *n* and the total number of collagen fibrils *n*_*c*_ is:

73 $$ n = n_{c} p(l;\bar{l},s), \quad l > l_{0} $$

where $p(l;\bar{l},s)$ is the probability of finding fibrils with a length of $l \ge l_{i} .$ Lake and Armeniades [49] regarded that a Gaussian function is reasonable for estimating this probability:

74 $$ p(l;\bar{l},s) = \frac{1}{{s\sqrt {2\pi } }}\exp \left[ - \frac{1}{2}\left(\frac{{l - \bar{l}}}{s}\right)^{2} \right] $$

The initial length *l*_*i*_ obeys the following expression:

75 $$ l_{i} (l;\bar{l},s) = \int_{{l_{0} }}^{l} x p(x;\bar{l},s)dx $$

where *x* is an integration variable. Substituting Eqs. (75) into (72), and considering Eq. (73), yields:

76 $$ F_{t} = F_{elastin} + F_{collagen} = n_{e} E_{e} (l - l_{0} ) + n_{c} E_{c} [l - l_{i} (l;\bar{l},s)] $$

The Lagrangian (engineering or nominal) stress, $\sigma_{0} = {{F_{t} } \mathord{\left/ {\vphantom {{F_{t} } {A_{0} }}} \right. \kern-0pt} {A_{0} }} ,$ can be calculated from Eq. (76) by dividing *A*_0_ (cross-section of a specimen at load-free state), and then the stress is written as:

77 $$ \sigma_{0} = E_{low} (\lambda - 1) + E_{high} [\lambda - l_{i} (l;\bar{l},s)/l_{0} ] $$

where *E*_*low*_ and *E*_*high*_ are the low- and high-strain Young’s moduli of the whole tissue, respectively. *E*_*low*_ = *n*_*e*_*E*_*e*_*l*_0_/*A*_0_ and *E*_*high*_ = *n*_*c*_*E*_*c*_*l*_0_/*A*_0_. The parameters $\bar{l}$ and *s* are determined by tissue microstructure and can be obtained from histological observations. For the bovine aorta, $\bar{l}/l_{0} \approx 1.5.$

The predicted stress–extension curves for the defatted and native tissues obtained using the model in Eq. (77) are compared with the experimental data in Fig. 10b. The comparison shows that the model is quite reasonable.

### Updated Recruitment Constitutive Models

In the light of the model above, a structural theory for homogenous biaxial stress–strain relations in flat collagen tissues was proposed in [50]. It was supposed that the tissues are composed of fiber networks to bear an applied load. A tensile test specimen contains a large number of fibers, *n*. Each fiber is purely elastic and has the same average cross-section *a*. The initial length *l*_*i*_ of these fibers is distributed around a mean length, $\bar{l} ,$ with a standard deviation *s*. The retractive force at a length *l* caused by a load applied at both ends of a specimen is written as [50]:

78 $$ F(l) = \int_{{l_{0} }}^{l} {ak\Bigg(\frac{l}{{l_{i} }}} - 1\Bigg)\frac{n}{{s\sqrt {2\pi } }}\exp \left[ - {\frac{1}{2}} \Bigg(\frac{{l_{i} - \bar{l}}}{s}\Bigg)^{2} \right]dl_{i} = \frac{akn}{{s\sqrt {2\pi } }}\int_{{l_{0} }}^{l} {\Bigg(\frac{l}{{l_{i} }}} - 1\Bigg)\exp \left[ - {\frac{1}{2}} \Bigg(\frac{{l_{i} - \bar{l}}}{s}\Bigg)^{2} \right]dl_{i} $$

where *l*_*i*_ is the initial fiber length.

Accordingly, the Lagrangian stress, $\sigma_{0} = {{F(l)} \mathord{\left/ {\vphantom {{F(l)} {A_{0} }}} \right. \kern-0pt} {A_{0} }} ,$ can be obtained from Eq. (78) by dividing both sides of the equation by *A*_0_ (cross-section of specimen at load-free state) as:

79 $$ \sigma_{0} (\lambda ) = \frac{{bl_{0} }}{{s\sqrt {2\pi } }}\int_{1}^{\lambda } {\Bigg(\frac{\lambda }{{\lambda_{i} }}} - 1\Bigg)\exp \left[ - {\frac{1}{2}} \left(\frac{{\lambda_{i} - {{\bar{l}} \mathord{/ {\vphantom {{\bar{l}} {l_{0} }}}\kern-0pt} {l_{0} }}}}{{{s \mathord{/ {\vphantom {s {l_{0} }}}  \kern-0pt} {l_{0} }}}}\right)^{2}\right]d\lambda_{i} $$

where *λ* = *l*/*l*_0_ and *λ*_*i*_ = *l*_*i*_/*l*_0_ are the stretches, and $b = akn/A_{0}$ is a constant. The model parameters *b*, $\bar{l} ,$ and *s* can be determined from experimental data.

The force–strain relation for maturing rat skin was modeled in [51] using a concept similar to that in [49]. The rat skin was considered to be a collagen fiber network only. The fibers have linear elasticity and a wavy pattern under the load-free condition. When stretched, the fiber become straightened by a recruitment function *R*(*l*), which is a normal Gaussian function with a mean value $\bar{l}$ and a standard deviation *s*, i.e.:

80 $$ R(l) = \frac{1}{{s\sqrt {2\pi } }}\exp \left[ - {\frac{1}{2}} \left(\frac{{l - \bar{l}}}{s}\right)^{2} \right] $$

A specimen was stretched to *l*_1_ from the initial length *l*_0_ (stress-free). When it is elongated further by increment Δ*l*_1_ starting from *l*_1_, the percentage of fibers recruited is approximately:

81 $$ P(l_{1} ) = R(l_{1} )\Delta l_{1} $$

The force generated by this increment is the product of *P*(*l*_1_), strain Δ*l*_1_/*l*_1_, and stiffness *k*, and is expressed by:

82 $$ F_{1} = k\frac{{\Delta l_{1} }}{{l_{1} }}R(l_{1} )\Delta l_{1} $$

Let *l*_2_ = *l*_1_ + Δ*l*_1_; then, the percentage of new fibers recruited because of new increment Δ*l*_2_ is $P(l_{2} ) = R(l_{2} )\Delta l_{2} ,$ and the new retractive force generated is:

83 $$ F_{2} = k\frac{{\Delta l_{1} }}{{l_{1} }}R(l_{1} )\Delta l_{1} + k\frac{{\Delta l_{2} }}{{l_{1} }}R(l_{1} )\Delta l_{1} + k\frac{{\Delta l_{2} }}{{l_{1} }}R(l_{2} )\Delta l_{2} $$

Equations (82) and (83) can be expressed in summation form as:

84 $$ F_{n} = k\sum\limits_{i = 1}^{n} {\Delta l_{i} \sum\limits_{j = 1}^{i} {\frac{{R(l_{j} )}}{{l_{j} }}\Delta l_{j} } } $$

If the number of fibers *n* is very large, the summation can be written in integral form:

85 $$ F = k\int_{{l_{1} }}^{l} {\int_{{l_{1} }}^{x} {\frac{R(y)}{y}} } dydx $$

The parameters *k*, $\bar{l} ,$ and *s* can be determined from experimental data for load and stretch. This author seems to be the first person proposing the recruitment concept for collagen fibers.

For the human scapholunate ligament in wrists, Nikolopoulos et al. [84]. proposed the following mathematical model to estimate stress and fit their experimental data:

86 $$ \sigma = E_{\hbox{max} } \left( {\lambda - \frac{1}{{\lambda^{2} }}} \right)\frac{1}{{s\sqrt {2\pi } }}\int_{1}^{x} {\exp \left[ { - \frac{1}{2}\left( {\frac{{x - \overline{\lambda } }}{s}} \right)^{2} } \right]} dx $$

where *E*_max_ is the maximum obtained Young’s modulus in the experiment, and $\overline{\lambda }$ and *s* are the mean value and standard deviation of initial fiber length *λ*_*i*_, respectively. This equation is different from Eq. (79) or (85), and needs to be investigated further.
